# Intestinal microbiome analyses identify melanoma patients at risk for checkpoint-blockade-induced colitis

**DOI:** 10.1038/ncomms10391

**Published:** 2016-02-02

**Authors:** Krista Dubin, Margaret K. Callahan, Boyu Ren, Raya Khanin, Agnes Viale, Lilan Ling, Daniel No, Asia Gobourne, Eric Littmann, Curtis Huttenhower, Eric G. Pamer, Jedd D. Wolchok

**Affiliations:** 1 Infectious Diseases Service, Department of Medicine, Memorial Sloan Kettering Cancer Center, 1275 York Avenue, New York, New York 10065, USA; 2 Lucille Castori Center for Microbes, Inflammation and Cancer, Memorial Sloan Kettering Cancer Center, New York, New York 10065, USA; 3 Weill Cornell Graduate School of Medical Sciences of Cornell University, New York, New York 10065, USA; 4 Weill Cornell Medical College, New York, New York 10065, USA; 5 Melanoma and Immunotherapy Service, Department of Medicine, Memorial Sloan Kettering Cancer Center, New York, New York 10065, USA; 6 Department of Biostatistics, Harvard School of Public Health, Boston, Massachusetts 02115, USA; 7 Computational Biology Program, Sloan-Kettering Institute, New York, New York 10065, USA; 8 Genomics Core Laboratory, Sloan-Kettering Institute, New York, New York 10065, USA; 9 Microbial Systems and Communities, Genome Sequencing and Analysis Program, The Broad Institute, Cambridge, Massachusetts 02142, USA; 10 Immunology Program, Sloan-Kettering Institute, New York, New York 10065, USA; 11 Ludwig Center for Cancer Immunotherapy, Memorial Sloan Kettering Cancer Center, New York, New York 10065, USA

## Abstract

The composition of the intestinal microbiota influences the development of inflammatory disorders. However, associating inflammatory diseases with specific microbial members of the microbiota is challenging, because clinically detectable inflammation and its treatment can alter the microbiota's composition. Immunologic checkpoint blockade with ipilimumab, a monoclonal antibody that blocks cytotoxic T-lymphocyte-associated antigen-4 (CTLA-4) signalling, is associated with new-onset, immune-mediated colitis. Here we conduct a prospective study of patients with metastatic melanoma undergoing ipilimumab treatment and correlate the pre-inflammation faecal microbiota and microbiome composition with subsequent colitis development. We demonstrate that increased representation of bacteria belonging to the Bacteroidetes phylum is correlated with resistance to the development of checkpoint-blockade-induced colitis. Furthermore, a paucity of genetic pathways involved in polyamine transport and B vitamin biosynthesis is associated with an increased risk of colitis. Identification of these biomarkers may enable interventions to reduce the risk of inflammatory complications following cancer immunotherapy.

## Abstract (web summary)

A subset of cancer patients treated with immune checkpoint blockade develops colitis. Here the authors show that lower abundance of Bacteroidetes and vitamin B biosynthetic modules in fecal samples of melanoma patients can predict their susceptibility to colitis following anti-CTLA-4 treatment.

## Graphical abstract

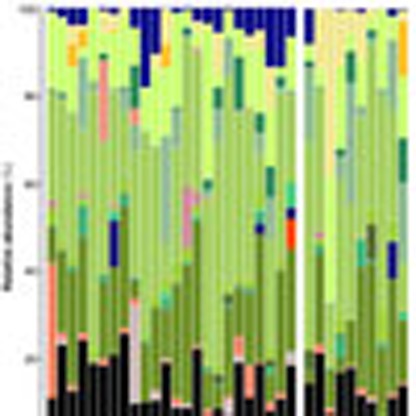

Complex microbial communities, known as the microbiota, colonize the mammalian intestine and contribute to the host's health^1^,^2^. Healthy individuals harbour distinct microbial populations in their intestinal tract that vary markedly in composition^3^,^4^. The complexity and plasticity of the intestinal microbiota represent a significant challenge to the host's immune defenses, which must balance immune tolerance of beneficial microbes with inflammatory responses against pathogens. Certain bacterial species are essential for maintaining a tolerogenic state in the mucosa. Microbial species from a variety of genera such as *Bacteroides*, *Clostridium* and *Faecalibacterium* can induce the expansion of T-regulatory cells or stimulate the production of anti-inflammatory cytokines^5^,^6^,^7^. Identifying the microbial species that promote homeostasis or drive inflammation has remained a challenge in the clinical context, in particular with chronic inflammatory conditions such as inflammatory bowel disease. Certain bacteria preferentially expand following inflammation, which alters the microbiota's composition^8^,^9^,^10^. Thus, as most patients seek medical attention after inflammation has developed, it is difficult to define the microbiota composition that precedes the development of colitis.

Ipilimumab, a monoclonal antibody that blocks the co-inhibitory molecule cytotoxic T-lymphocyte-associated antigen-4 (CTLA-4), is an immunomodulatory therapy that provides effective treatment against metastatic melanoma^11^. Inhibition of CTLA-4 signalling dampens negative regulation of T cells, thereby enhancing anti-tumour responses. Within the first 16 weeks of treatment, roughly one-third of recipients develop intestinal inflammation as a result of mucosal immune dysregulation^12^,^13^,^14^. The high incidence of colitis in patients receiving ipilimumab provides an opportunity to characterize the colonic microbiota before the development of intestinal inflammation. Herein we use next-generation metagenomic sequencing to identify biomarkers that are associated with resistance to new-onset, immune-mediated colitis in the context of immune checkpoint-blockade therapy. Increased faecal abundance of the Bacteroidetes phylum and three of its families (Bacteroidaceae, Rikenellaceae and Barnesiellaceae), as well as microbial genetic pathways involved in polyamine transport and B vitamin biosynthesis, are correlated with resistance to the development of colitis following CTLA-4 blockade. This study provides a novel view of the intestinal microbiota before the development of colitis and offers insight into preventive treatments for patients at risk of adverse inflammatory events following immunologic checkpoint blockade.

## Results

### Colitis development in patients following CTLA-4 blockade

**Figure 1:**
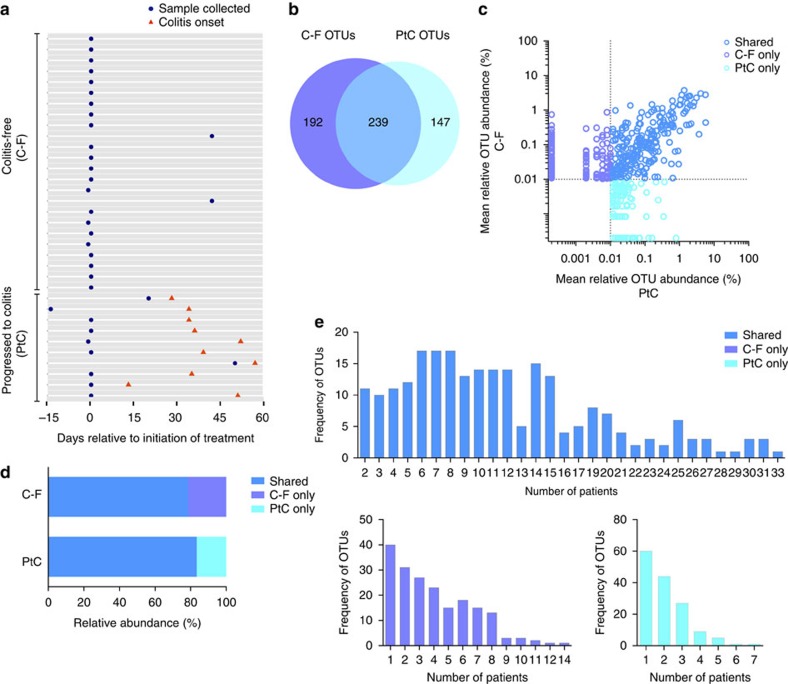
C-F and PtC patients harbour distinct microbial populations. (**a**) Faecal sample collection (blue circle) and the onset of colitis (red triangle) are shown at the indicated time points during treatment with ipilimumab, a monoclonal antibody that blocks CTLA-4 signalling. Dates are relative to first dose of treatment. An average abundance >0.01% was used as the threshold for considering an OTU to be present within faecal samples in either patient group (OTUs, *n*=578); OTUs at an abundance of ≤0.01% were not considered to be present in the patients' microbiota. Using this definition, we calculated (**b**) the number of OTUs present in C-F patients only (purple), PtC patients only (turquoise) or shared between the patient groups (blue) in a scaled Venn diagram. (**c**) The mean relative abundance of OTUs, (**d**) total abundance of OTUs, (**e**) distribution of OTUs that are present in C-F patients only, PtC patients only or shared between the patient. C-F patients, *n*=24; PtC patients, *n*=10. OTUs, operational taxonomic units.

We analysed the intestinal microbiota of 34 patients enrolled in a prospective study to correlate pre-colitis faecal composition with the subsequent development of checkpoint-blockade-induced colitis. The patients in this study ranged in age between 28 and 85 years, and were diagnosed with metastatic melanoma. In general, faecal samples were obtained from patients before the first dose of ipilimumab (30/34), although in two patients who subsequently developed inflammation and two patients who remained inflammation free, samples were obtained after initiation of ipilimumab, but before the onset of colitis. Of the 34 patients, 10 were diagnosed with gastrointestinal inflammation between 13 and 57 days after initiation of ipilimumab (Fig. 1a). Patients who did not develop gastrointestinal inflammation following CTLA-4 blockade are herein referred to as colitis free (C-F) and patients who experienced inflammatory complications after CTLA-4 blockade are referred to as having progressed to colitis (PtC). Patients were assigned a colitis score by retrospective chart review, which ranged from no diarrhoea (score 0) in C-F patients to severe colitis (score 4) in PtC patients (Supplementary Table 1). There were no differences in age or gender between C-F and PtC patients (Supplementary Table 1). In addition, 40% of PtC patients and 50% of C-F patients were treated with systemic cancer therapy administered either before (14/16) or both before and during (2/16; both C-F) ipilimumab treatment course (Supplementary Table 1).

### Composition of the intestinal microbiota before colitis

To characterize the intestinal microbial composition before immune-mediated colitis, faecal samples were submitted for bacterial microbiota profiling using 16S ribosomal RNA sequencing on the Illumina MiSeq platform. C-F and PtC patients harboured intestinal microbiota with similarly complex microbial populations and shared 239 of 578 operational taxonomic units (OTUs, defined at a 97% sequence similarity; Fig. 1b). These 239 shared OTUs represent 79% and 83% of the total OTU abundance in the C-F and PtC patient groups, respectively (Fig. 1c,d), and many were present in 5 or more patients (Fig. 1e). In contrast, OTUs that were either associated with only C-F or only PtC patients were generally detected in fewer than five patients (Fig. 1e). The OTUs that are shared between the two patient groups are more evenly distributed. Roughly 20% of shared OTUs are found in over half of all patients (Fig. 1e). These differences in the distribution of OTUs among the patient groups did not have a significant impact on the overall biodiversity (Supplementary Fig. 1a,b). Microbial richness, reflecting the number of unique phylotypes within a given sample, was found to be similar between patients who developed CTLA-4 blockade-induced colitis and those who remained C-F (Supplementary Fig. 1c,d).

**Figure 2:**
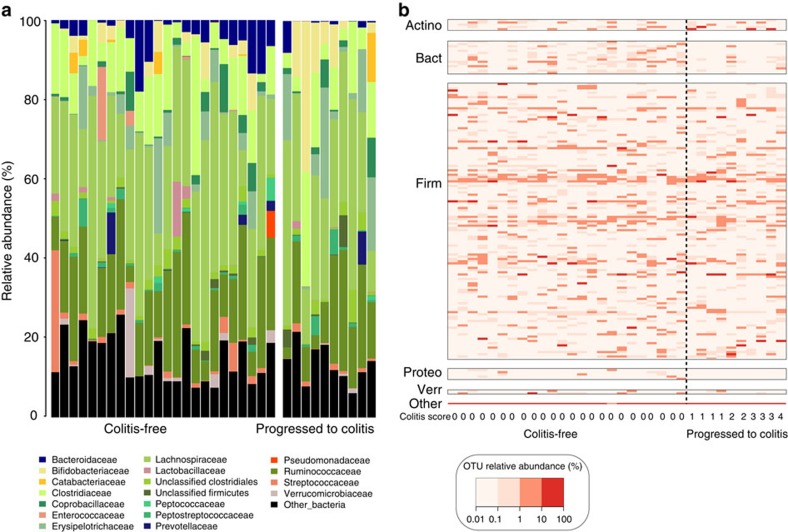
Composition of the intestinal microbiota between C-F and PtC patients. (**a**) OTUs with an average abundance >0.01% within either patient group were classified at the family taxonomic level. Families with an average abundance of 2.5% across all samples or an abundance of greater than 5% in a single sample are plotted. Each bar represents the faecal microbial composition of one patient. (**b**) The relative abundances of the 146 bacterial OTUs in C-F and PtC patients represented in a heat map. OTUs plotted were present at a mean abundance of ≥0.1%. Patients are ordered by CTCAE-based colitis score. C-F patients, *n*=24; PtC patients, *n*=10. OTUs, operational taxonomic units; Actino, Actinobacteria; Bact, Bacteroidetes; Firm, Firmicutes; Proteo, Proteobacteria; Verr, Verrucomicrobia.

To examine the intestinal microbiota structure between patient groups, we plotted the relative frequencies of bacterial phylotypes at the taxonomic level of family for each patient. Although C-F and PtC patients shared many bacterial taxa that belong to the Firmicutes phylum, C-F patients harbour a greater proportion of microbes within the Bacteroidaceae family (Fig. 2a). Our taxonomic classifications based on 16S sequencing data were similar to those found when applying MetaPhlAn to shotgun metagenomic sequencing data on a subset of this patient cohort, although the proportions of some bacterial phyla vary between the two methods (Supplementary Fig. 2). To illustrate the microbiota composition with finer resolution, we plotted the relative abundances of 146 OTUs at 0.1% or greater mean abundance. Although broad swaths of the microbiota are found at similar frequencies between patients, microbes within the Bacteroidetes phylum are underrepresented in the patients who developed new-onset, immune-mediated colitis (Fig. 2b).

### Members of Bacteroidetes phylum associated with resistance

**Figure 3:**
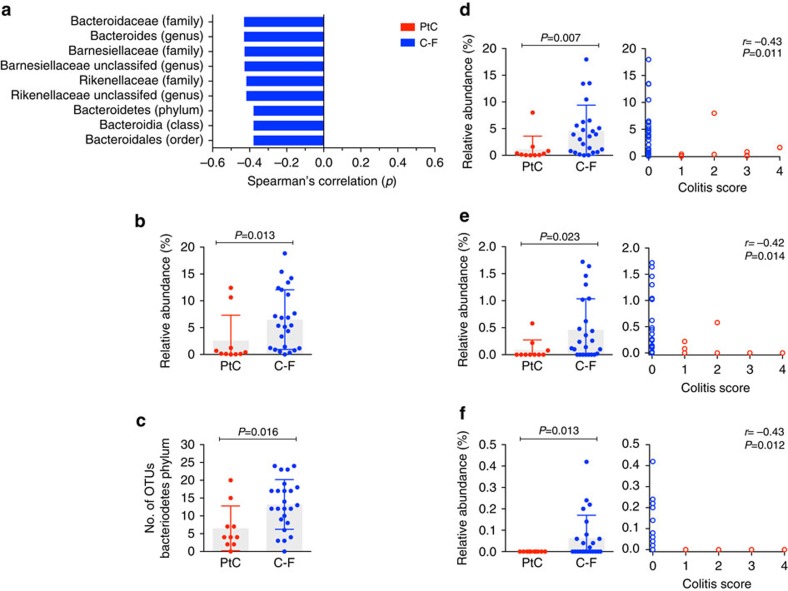
Increased abundance of the Bacteroidetes phylum and select families correlates with protection from colitis. OTUs with an average abundance >0.01% within either patient group were binned at different levels of taxonomic classification (phylum, class, order, family and genus). (**a**) Correlation of bacterial phylotypes to CTCAE-based colitis score by Spearman analysis. Taxa with *P*-values <0.05 are plotted. (**b**) Relative abundance of the phylum Bacteriodetes in PtC and C-F patients. (**c**) The number of OTUs assigned to the Bacteroidetes phylum in each patient group. Relative abundances of the families (**d**) Bacteroidaceae, (**e**) Rikenellaceae and (**f**) Barnesiellaceae within the Bacteroidetes phylum in PtC and C-F patients. *P*-values were determined by Mann–Whitney test. Height of bar represents the mean, error bars represent s.d. C-F patients, *n*=24; PtC patients, *n*=10. *r*, *ρ* coefficient; OTUs, operational taxonomic units.

To further explore the relationship between specific bacterial members of the intestinal microbiota and development of CTLA-4 blockade-induced colitis, we stratified patients by their severity of inflammation and performed a Spearman's rank correlation test on the relative abundances of bacterial species grouped at different taxonomic levels. This analysis revealed that taxa within the Bacteroidetes phylum are more prevalent in C-F patient samples (Fig. 3a). The relative abundance of OTUs classified as Bacteroidetes, as well as the number of OTUs assigned to the phylum, were significantly higher in C-F patients (Mann–Whitney test, *P*<0.05; Fig. 3b,c). Within the Bacteroidetes phylum, the prevalence of Bacteroidaceae, Rikenellaceae and Barnesiellaceae is significantly more abundant in patients resistant to ipilimumab-induced colitis (Mann–Whitney test, *P*<0.01, *P*<0.05 and *P*<0.05, respectively; Fig. 3d–f). Although the composition of the intestinal microbiota can vary over the lifetime of an individual, there was no association between patients' age and the abundance of Bacteroidetes (Supplementary Fig. 3).

### Specific microbial modules associated with protection

**Figure 4:**
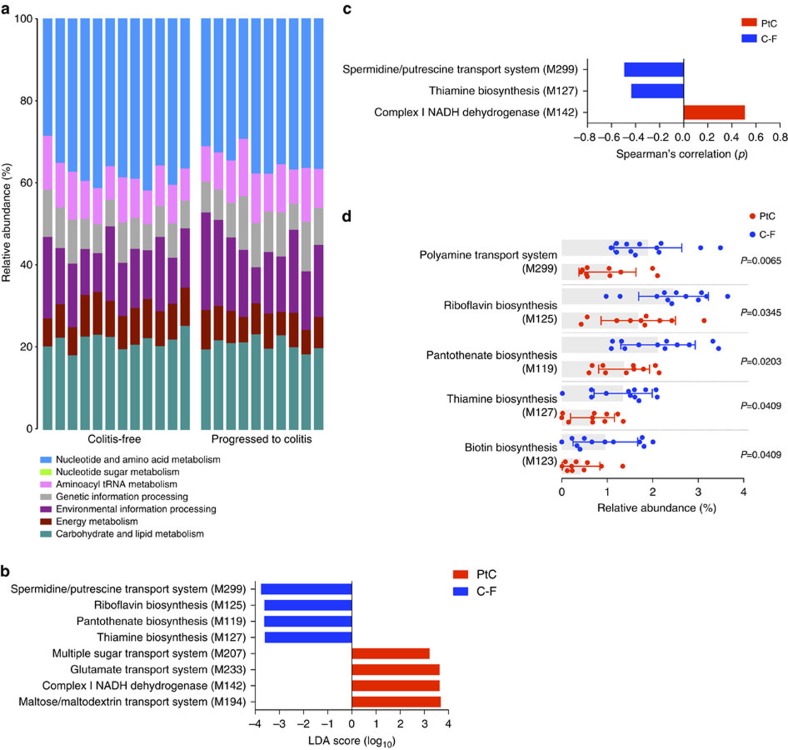
Bacterial modules involved in polyamine transport and vitamin B synthesis are associated with resistance to colitis. (**a**) Relative abundance of 102 microbial KEGG modules in C-F and PtC patients. (**b**) Association of genetic modules with colitis status by LEfSe analysis. Modules with a linear discriminant analysis (LDA) score >3 are plotted. (**c**) Correlation of genetic modules to colitis score by Spearman analysis. Modules with *P*-values <0.05 are plotted. (**d**) Relative abundances of modules associated with C-F patients. *P*-values were determined by Mann–Whitney test. Height of bar represents mean, error bars represent s.d. C-F patients, *n*=12; PtC patients, *n*=10.

To evaluate the genetic pathways that may play a role in the development of immune-mediated colitis, we performed shotgun metagenomic sequencing on the 10 PtC and 12 C-F patient faecal samples. Sequencing reads were processed using HUMAnN and assigned to KEGG (Kyoto Encyclopedia of Genes and Genomes) modules. The microbial modules that comprised patients' intestinal microbiota were broadly similar between C-F and PtC patients (Fig. 4a). We conducted a univariate test for associations between colitis status and the 102 microbial modules using linear discriminant analysis effect size (LEfSe) analysis. We found that the spermidine/putrescine polyamine transport system and three modules involved in the biosynthesis of B vitamins (riboflavin (B2), pantothenate (B5) and thiamine (B1)) were more abundant in C-F patients (Fig. 4b). After stratifying samples by colitis status, a Spearman analysis identified the polyamine transport system and thiamine (B1) modules as correlated with resistance to the development of colitis (Fig. 4c). The relative abundances of the aforementioned pathways are significantly enriched in C-F patient samples (Mann–Whitney test, *P*<0.05 for all modules; Fig. 4d). In addition, a module involved in the biosynthesis of the biotin (vitamin B7) was found in greater abundance in C-F patients (Mann–Whitney test, *P*<0.05).

### Select bacterial modules can identify colitis patients

**Figure 5:**
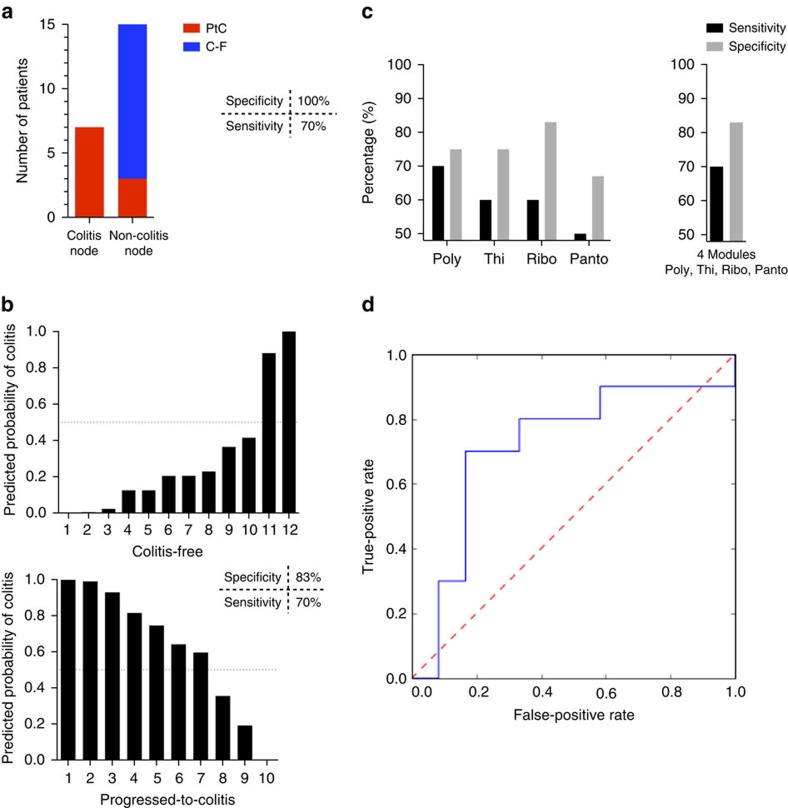
Predictive accuracy of bacterial modules to identify patients who develop colitis. (**a**) The recursive partitioning algorithm was used to construct a classification tree, based on the abundance of the polyamine transport module. (**b**) Leave-one-out cross-validation of the probit regression analysis predicts the probability of colitis using four modules associated with colitis resistance: polyamine transport system, thiamine biosynthesis, riboflavin biosynthesis and pantothenate biosynthesis. One patient is represented per column. Specificity and sensitivity calculated based on a probability threshold of 50%. (**c**) The sensitivity and specificity of each module to predict patients' colitis status by their faecal microbial samples was determined using a probability threshold of 50%, as compared with the four-module model. (**d**) Receiver operating characteristic (ROC) curve of the four-module model predicting colitis risk. ROC curve was created by calculating the true-positive rate and false-positive rate for 10,000 thresholds of the predicted probability of colitis between 0 and 1. True-positive rate represents the test sensitivity, calculated by: true positives/(true positives + false negatives). False-positive rate, which is given by 1−test specificity, is calculated by: false positives/(false positives + true negatives). Poly, polyamine transport system; Thi, thiamine biosynthesis; Ribo, riboflavin biosynthesis; Panto, pantothenate biosynthesis. C-F patients, *n*=12; PtC patients, *n*=10.

We next sought to assess the predictive accuracy of these microbial modules in determining a patient's risk for colitis. Using the recursive partitioning machine learning algorithm to form a classification tree based on the 102 modules, samples were successfully classified as from either PtC or C-F patients using the relative abundance of polyamine transport system module alone (Fig. 5a). Samples with >1% relative abundance of the polyamine transport system were binned as C-F (Supplementary Fig. 4). Of the PtC patients, 7 samples were appropriately identified, whereas 3 samples were misclassified as C-F, resulting in a sensitivity of 70% and specificity of 100% (Fig. 5a). Performing a leave-one-out cross-validation of the probit regression analysis based on 4 of the modules associated with C-F patients, we correctly predicted the colitis status for 10 out of 12 C-F patients and 7 out of 10 PtC patients, at a probability threshold of 50% (Fig. 5b). This model results in a sensitivity of 70% and specificity of 83%. For this regression analysis, we selected the four modules associated with resistance to colitis that were identified in the Spearman and LEfSe analyses, which include the polyamine transport system module and modules involved in the biosynthesis of vitamins riboflavin (B2), pantothenate (B5) and thiamine (B1). These modules performed better in combination than any individual module (Fig. 5c). Taken together, our four-module analysis predicts colitis risk with good accuracy as demonstrated by receiver operating characteristic curve, for which there are no other known biomarkers presented in the literature to use for comparison (Fig. 5d)^12^,^15^. In all, these analytical models identify bacterial pathways that may confer resistance to colitis and serve as biomarkers for patients at high risk of developing CTLA-4 blockade-induced colitis.

## Discussion

Our study is the first to characterize the intestinal microbiota of patients before the development of intestinal inflammation and has identified microbiota-associated biomarkers that correlate with protection against CTLA-4 blockade-associated colitis.

Our finding that members of the Bacteroidetes phylum are enriched in colitis-resistant patients is consistent with a proposed immunomodulatory role of these commensal bacteria. Bacteroidetes represents one of the major phyla of the human colonic microbiota and its members can limit inflammation by stimulating T-regulatory cell differentiation^16^,^17^. In addition, we show that the presence of microbiota-associated modules for bacterial polyamine transport system and the biosynthesis of thiamine, riboflavin and pantothenate can accurately assess a patient's risk of developing colitis following CTLA-4 blockade. The microbiota plays an important role in the endogenous synthesis of water-soluble B vitamins^18^,^19^,^20^. Thiamine and riboflavin concentrations are significantly reduced in the blood of patients with Crohn's disease and the levels of bound pantothenate in the colonic mucosa decrease with the progression of inflammatory bowel disease^21^,^22^. An innate-like T-cell population known as mucosal-associated invariant T cells are activated *in vitro* by riboflavin metabolites^23^. These cells accumulate in the inflamed mucosa of Crohn's disease patients, although it is not yet known whether their interleukin-17-skewed cytokine expression exacerbates or limits inflammation^24^. Polyamines, which are small cationic amines that can be exported from bacteria cells through the spermidine and putrescine transport system (potA, B, C and D), play an anti-inflammatory role by promoting colonic epithelial cell proliferation to maintain the epithelial barrier^25^. In patients with active colitis, levels of orthinine decarboxylase, the enzyme involved in polyamine synthesis, are lower than in control patients, but it remains unclear whether reduced polyamine levels contribute to colitis development or progression^26^,^27^. Additional studies are required to further explore whether a reduced capacity for microbe-mediated production of B vitamins and polyamine transport lowers the threshold for initiation of immune-mediated colitis.

Other checkpoint-blockade therapies, such as anti-PD1, can lead to adverse gastrointestinal events^28^. In the case of anti-PD-L1 and anti-CTLA-4 blockade, as well as CpG-oligonucleotide immunotherapy and cyclophosphamide, therapeutic response is affected by the intestinal microbiota^29^,^30^,^31^,^32^. As with treatment efficacy, the microbiota may play a role in the development of immune-mediated, new-onset colitis in the context of other immunotherapies. Identification of biomarkers that predict the risk of developing colitis may help identify patients that are particularly susceptible to distinct forms of immunotherapy-induced inflammation, such as with CTLA-4 blockade, and may facilitate preemptive treatments.

## Methods

### Study patients and specimen collection

Thirty-four adults analysed in our study were diagnosed with metastatic melanoma and received ipilimumab at Memorial Sloan-Kettering Cancer Center. Subjects had no previous history of colitis and bowel resection, and had not received antibiotic treatment in the preceding 2 months. Patients were excluded from analysis if the first faecal sample was collected after the onset of colitis, if the patient did not receive ipilimumab, or if colitis status was unknown. Ipilimumab monotherapy was administered at a dose of 3 mg kg^−1^ every 3 weeks for up to four doses, with three exceptions. Patients #10 and #15 received vemurafenib before and during ipilimumab treatment as part of a clinical trial. Patient #13 was part of a blinded clinical trial and received ipilimumab at either 3 or 10 mg kg^−1^. A small number of patients (PtC, *n*=2; C-F, *n*=3) received additional doses of ipilimumab either as part of maintenance treatment (every 3 months) on a clinical study or as part of a second course of ipilimumab; however, the colitis cases documented here occurred before this additional dosing was administered. Systemic cancer treatment administered before (*n*=14) or both before and during (*n*=2) ipilimumab therapy was determined retrospectively on chart review by a single investigator (MKC). For 30 patients, faecal samples were collected from each participant before the first administration of ipilimumab; for 2 patients who developed gastrointestinal inflammation and 2 patients who remained inflammation free, samples were collected after the initiation of CTLA-4 blockade, but before onset of colitis. Toxicities were graded retrospectively by a single investigator (MKC) based on chart review using CTCAE, version 4.0, and grading on the terms diarrhoea and colitis. Patients were assigned a colitis score based on the following: no diarrhoea (score 0), grade 1 diarrhoea (score 1), grade 2 diarrhoea (score 2), grade 2 diarrhoea and/or grade 2 colitis (all 3 cases had both grade 2 diarrhoea and grade 2 colitis) (score 3), grade 3 diarrhoea and/or grade 3 colitis (1 case with both grade 3 diarrhoea and grade 3 colitis) (score 4). All participants provided written consent for specimen collection and analysis under the study protocol approved by the Memorial Sloan-Kettering Cancer Center Institutional Review Board.

### DNA extraction

Each faecal sample was immediately snap-frozen at −80 °C and subsequently subjected to bead beating and phenol chloroform extraction for DNA purification. Samples were resuspended in 500 μl of an extraction buffer (200 mM Tris pH 8.0/200 mM NaCl/20 mM EDTA), 200 μl of 20% SDS, 500 μl of phenol:chloroform:isoamyl alcohol (24:24:1) and 500 μl of 0.1-mm diameter zirconia/silica beads (BioSpec Products). For 2 min, cells were lysed by mechanical disruption using a bead beater. DNA was extracted in a phenol/chloroform/isoamyl solution twice and precipitated with ethanol and sodium acetate. DNA was resuspended in 200 μl of TE buffer containing 100 μg ml^−1^ RNase and further purified using QIAmp Mini Spin Columns (Qiagen). After eluting the sample in 100 μl of distilled water, double-stranded DNA was quantified.

### 16S rRNA gene amplification and multiparallel sequencing

The V4–V5 region of the 16S rRNA gene was amplified and sequenced on an Illumina MiSeq platform. For each faecal sample, replicate PCR reactions were performed using modified universal bacterial primers designed to amplify the V4-V5 16S rRNA region: 563F (59-nnnnnnnn-NNNNNNNNNNNN-AYTGGGYDTAAAGN G-39) and 926R (59-nnnnnnnn-NNNNNNNNNNNN-CCGTCAATTYHTTTR AGT-39). Each reaction contained 50 ng of purified DNA, 0.2 mM dNTPs, 1.5 μM MgCl_2_, 1.25 U Platinum TaqDNA poly-merase, 2.5 μl of 10 × PCR buffer and 0.2 μM of each primer. A unique 12-base Golay barcode (Ns) preceded the primers for sample identification after pooling amplicons. One to eight additional nucleotides were added before the barcode to offset the sequencing of the primers. Cycling conditions were the following: 94 °C for 3 min, followed by 27 cycles of 94 °C for 50 s, 51 °C for 30 s and 72 °C for 1 min, where the final elongation step was performed at 72 °C for 5 min. Replicate PCRs were combined and were subsequently purified using the Qiaquick PCR Purification Kit (Qiagen) and Qiagen MinElute PCR Purification Kit. Using the Illumina TruSeq Sample Preparation procedure, PCR products were quantified and pooled at equimolar amounts before Illumina barcodes and adaptors were ligated on. The completed library was sequenced on an Ilumina Miseq platform according to the Illumina recommended protocol.

### Sequence analysis

Sequences were analysed using mothur version 1.31.1 (ref. 33). Sequences were aligned using the Silva reference alignment as a template and potentially chimeric sequences were eliminated using the UChime algorithm^34^. Five thousand sequences per patient were selected (mean 4,974, s.d. 150) and sequences with a distance-based similarity of ≥97% were grouped into OTUs using the furthest-neighbour algorithm. OTUs were classified using the Greengenes 16S rRNA reference database. OTU-based microbial diversity was estimated by calculating two diversity indices, Shannon and Inverse Simpson. OTU-based richness was determined by calculating the Chao richness estimate and constructing rarefaction curves. OTUs were grouped at different levels of classification (phylum, class, order, family and genus); at each level, OTUs that did not have a classification were grouped together by the highest available resolution (for example, at the genus level, an OTU classified as p__Bacteroidetes_c__Bacteroidia_o__Bacteroidales_ f__Barnesiellaceae_unclassifed would be grouped as f__Barnesiellaceae_unclassifed). Feature selection of the intestinal microbia's composition was performed on OTUs with an average abundance >0.01% in either patient group and grouped by phylotype.

### Shotgun sequencing and metabolic pathway reconstruction

Stool samples from all 10 PtC patients and 12 C-F patients were subjected to shotgun sequencing. C-F patient samples were chosen so that the full spectrum of Bacteroidetes phylum abundance was represented. Libraries were constructed with Illumina barcodes from the TruSeq DNA Sample Prep kit (Illumina) and reagents from KAPA Library Preparation kit (Kapa Biosystems), and then sequenced on an Illumina MiSeq platform using 2 × 250 nucleotide paired-end sequencing, according to the manufacturer's instructions. Sequencing reads were converted into relative abundances of microbial metabolic modules using HUMAnN^35^, the Human Microbiome Project metabolic reconstruction pipeline and mapped to the KEGG^36^. Relative species abundances were calculated by the MetaPhlAn pipeline^37^.

### Statistics

Statistical analyses of intestinal microbiota samples were performed using R Statistical Language (v3.1.1) and GraphPad Prism (version 6.0e) software packages. Unpaired Mann–Whitney rank sum test (two-tailed) was used for comparisons of continuous variables between two groups. Bar plots were used to represent the data's mean at the centre values, with error bars to indicate s.d. Spearman's rank correlation tests (two-tailed) were used to find significant correlations between two continuous variables. LEfSe was used to identify differentially abundant features between classes of samples^38^. Recursive partitioning to form classification trees was performed in R using the package rpart. Generalized linear model (using probit regression and the glm R-function) was constructed on all combinations of the following five modules associated with C-F patients: polyamine transport system (M299), riboflavin biosynthesis (M125), pantothenate biosynthesis (M119), thiamine biosynthesis (M127) and biotin biosynthesis (M123). Leave-one-out cross-validation was used to compute model sensitivity and specificity. The reported model provided the best total sensitivity plus specificity. Unadjusted *P*-values <0.05 were considered to be significant for the Mann–Whitney rank sum test and Spearman's rank correlation tests.

## Code availability

Code, written in R version 3.2.2, and instructions are provided in the Supplementary Software file.

## Additional information

**Accession codes:** 16S rRNA gene sequences and shotgun sequences (forward R1, reverse R2) analysed in this study have been deposited in the NCBI SRA database under the BioProject ID: PRJNA302832. The associated BioSamples' accession numbers are the following: SAMN04281019, SAMN04281020, SAMN04281021, SAMN04281022, SAMN04281023, SAMN04281024, SAMN04281025, SAMN04281026, SAMN04281027, SAMN04281028, SAMN04281029, SAMN04281030, SAMN04281031, SAMN04281032, SAMN04281033, SAMN04281034, SAMN04281035, SAMN04281036, SAMN04281037, SAMN04281038, SAMN04281039, SAMN04281040, SAMN04281041, SAMN04281042, SAMN04281043, SAMN04281044, SAMN04281045, SAMN04281046, SAMN04281047, SAMN04281048, SAMN04281049, SAMN04281050, SAMN04281051, SAMN04281052 || SAMN04281200, SAMN04281201, SAMN04281202, SAMN04281203, SAMN04281204, SAMN04281205, SAMN04281206, SAMN04281207, SAMN04281208, SAMN04281209, SAMN04281210, SAMN04281211, SAMN04281212, SAMN04281213, SAMN04281214, SAMN04281215, SAMN04281216, SAMN04281217, SAMN04281218, SAMN04281219, SAMN04281220, SAMN04281221, SAMN04281222, SAMN04281223, SAMN04281224, SAMN04281225, SAMN04281226, SAMN04281227, SAMN04281228, SAMN04281229, SAMN04281230, SAMN04281231, SAMN04281232, SAMN04281233, SAMN04281234, SAMN04281235, SAMN04281236, SAMN04281237, SAMN04281238, SAMN04281239, SAMN04281240, SAMN04281241, SAMN04281242, SAMN04281243.

**How to cite this article**: Dubin, K. *et al.* Intestinal microbiome analyses identify melanoma patients at risk for checkpoint-blockade-induced colitis. *Nat. Commun.* 7:10391 doi: 10.1038/ncomms10391 (2016).

## Supplementary Material

Supplementary Information Supplementary Figures 1-4 and Supplementary Table 1

Supplementary Software A readme.txt and R code
