# Supplementary material for: Intestinal microbiome analyses identify melanoma patients at risk for checkpoint-blockade-induced colitis
Source: Nat Commun. 2016 Feb 2;7:10391. doi: 10.1038/ncomms10391 (PMC4740747; doi:10.1038/ncomms10391)
Supplement: Supplementary Information — Supplementary Figures 1-4 and Supplementary Table 1 [file ncomms10391-s1.pdf]

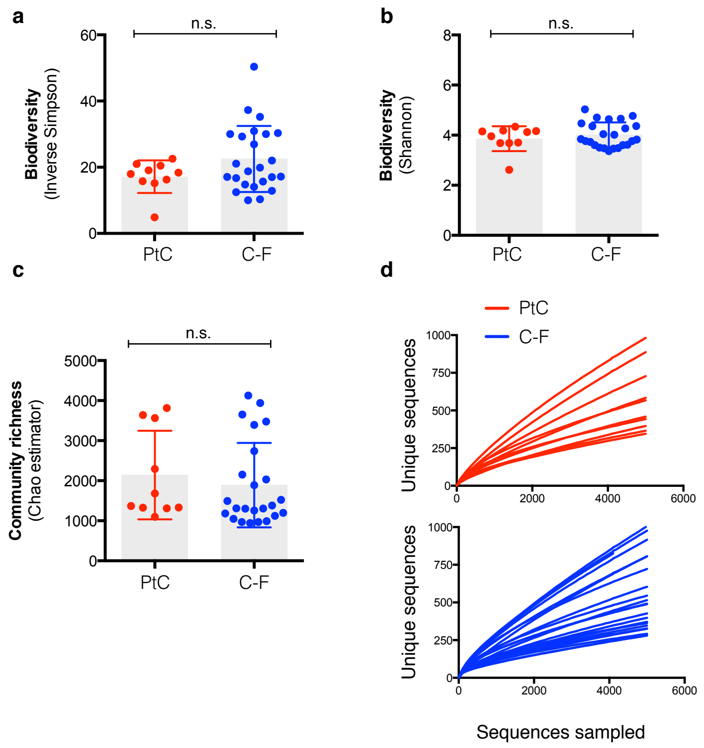

Supplementary Figure 1 | Colitis-Free and Progressed to Colitis patients have similar levels of intestinal microbial diversity. Biodiversity is estimated by a) the Inverse Simpson index and b) the Shannon index for each patient sample. Richness is measured by c) the Chao estimator and d) rarefaction curves. Mann-Whitney tests were used to compare microbial diversity parameters between the patient groups. Height of bar represents the mean, error bars represent standard deviation. C-F, Colitis-Free; PtC, Progressed to Colitis. C-F patients, n=24; PtC patients, n=10. n.s., not significant.

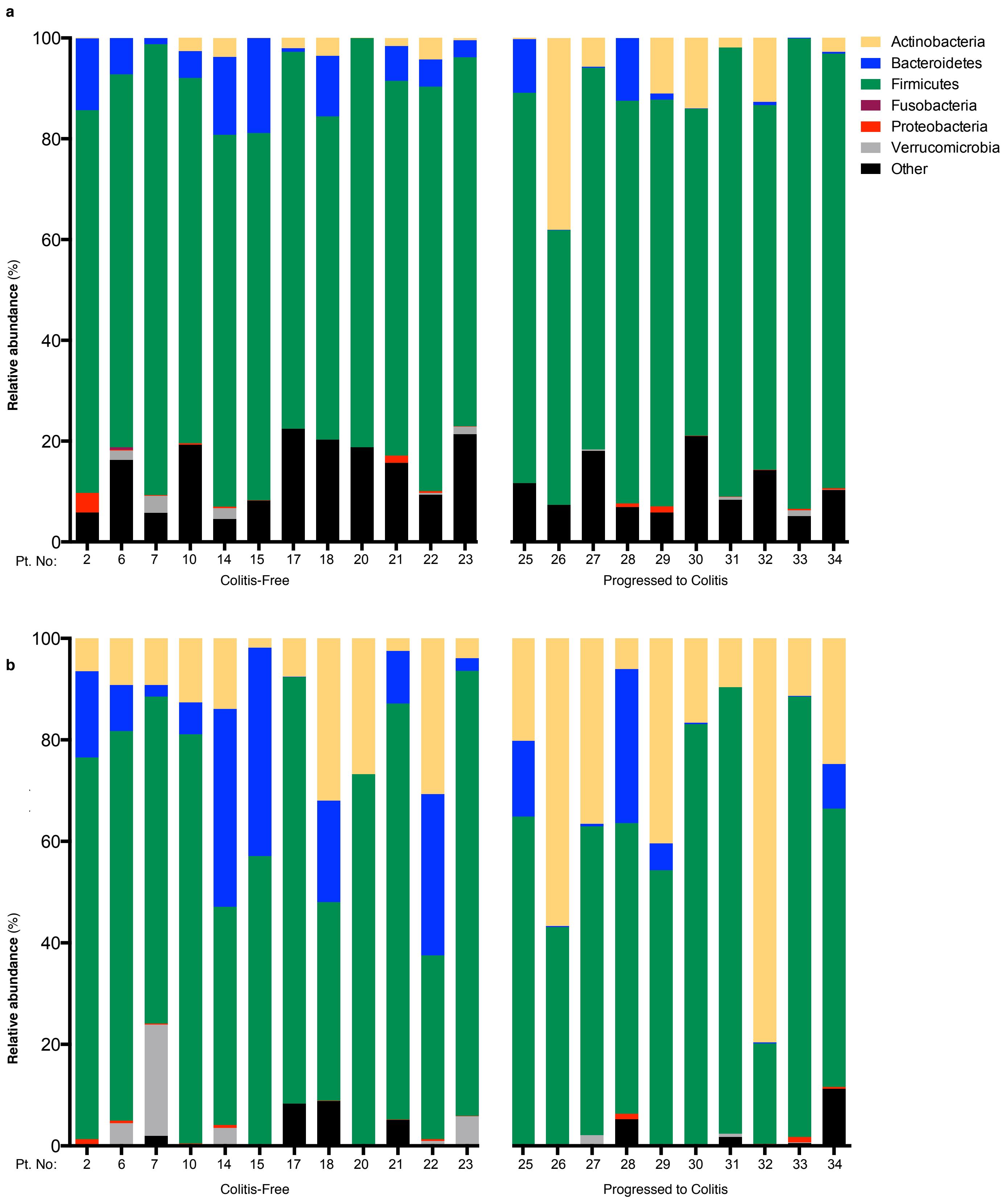

Supplementary Figure 2 | Accuracy of bacterial taxonomic classification. a) 16s sequencing reads were clustered into OTUs by 97% sequencing homology. Relative abundances of OTUs that met our defined threshold (of an average abundance greater than 0.01% in either patient group) were plotted by phylum. Bar plots were constructed only for those patients whose samples were subjected to shotgun metagenomic sequencing. b) Relative abundances of shotgun metagenomic sequencing reads were calculated using MetaPhlAn and plotted by phylum. OTUs, Operational Taxonomic Units. C-F patients, n=12; PtC patients, n=10.

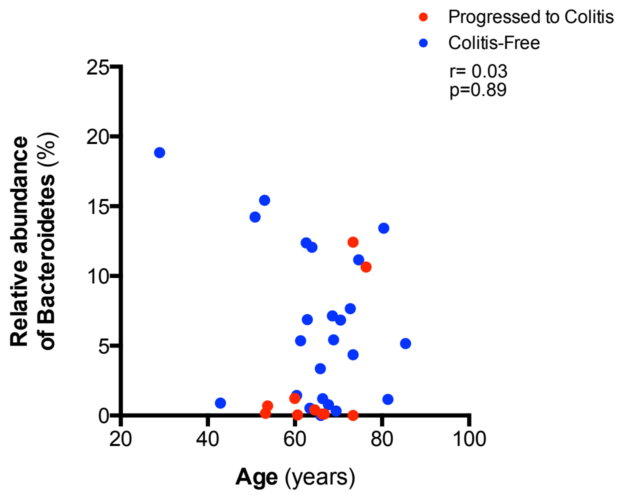

Supplementary Figure 3 | Correlation between patient age and relative abundance of Bacteroidetes phylum. Patient age was defined as of the first dose of ipilimumab. OTUs with an average abundance greater than 0.01% within either patient group were grouped by phylum. Spearman correlation test was performed on age and Bacterodietes abundance. r, Rho coefficient; p, p-value; OTUs, Operational Taxonomic Units. C-F patients, n=24; PtC patients, n=10.

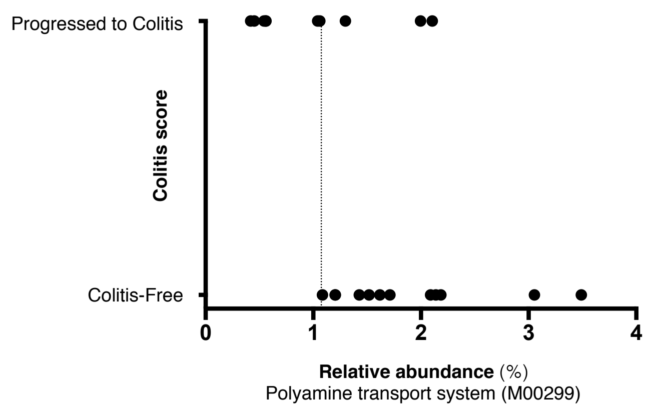

Supplementary Figure 4 | Module abundance of the polyamine transport system. The recursive partitioning algorithm estimated colitis status of patient samples based on polyamine transport system abundance alone. Samples with a module abundance of 1% or greater were classified as Colitis-Free by this algorithm. C-F patients, n=12; PtC patients, n=10.

Patient group	Patient no.	Colitis Score	Sex	Age	Prior Systemic Cancer Treatment(s)
Colitis-Free	1	0	M	62	Tyrosinase DNA vaccine (in adjuvant setting)
Colitis-Free	2	0	F	50	Adjuvant Interferon
Colitis-Free	3	0	M	80	-
Colitis-Free	4	0	M	74	Vemurafenib
Colitis-Free	5	0	F	72	-
Colitis-Free	6	0	M	68	-
Colitis-Free	7	0	F	66	-
Colitis-Free	8	0	M	70	-
Colitis-Free	9	0	F	73	Anti-gp75 monoclonal antibody IMC-20D7S; AZD6244; vemurafenib
Colitis-Free	10	0	F	61	*Vemurafenib
Colitis-Free	11	0	M	63	-
Colitis-Free	12	0	M	60	-
Colitis-Free	13	0	M	69	-
Colitis-Free	14	0	F	53	Vemurafenib
Colitis-Free	15	0	F	28	*Vemurafenib
Colitis-Free	16	0	M	85	-
Colitis-Free	17	0	F	67	-
Colitis-Free	18	0	M	63	Cisplatin + vinblastine + temozolomide
Colitis-Free	19	0	M	42	Nivolumab (in clinical trial)
Colitis-Free	20	0	F	65	Cisplatin + vinblastine + temozolomide + R04929097; vinblastine
Colitis-Free	21	0	M	62	-
Colitis-Free	22	0	M	68	Temozolomide
Colitis-Free	23	0	F	65	-
Colitis-Free	24	0	M	81	Temozolomide; AZD6224
Progressed to Colitis	25	2	M	76	Vaccine, GD2L-KLH with OPT-821
Progressed to Colitis	26	3	F	66	-
Progressed to Colitis	27	3	M	60	Adjuvant Interferon
Progressed to Colitis	28	4	M	53	-
Progressed to Colitis	29	3	M	53	Vemurafenib
Progressed to Colitis	30	1	F	59	Temozolomide
Progressed to Colitis	31	1	M	73	-
Progressed to Colitis	32	1	F	66	-
Progressed to Colitis	33	1	M	73	-
Progressed to Colitis	34	2	F	64	-

Supplementary Table 1 | Patient characteristics and sample collection. Colitis score was determined by retrospective chart review using CTCAE, with scores ranging from 0 (no diarrhea) to 4 (severe colitis). Patient age was defined as of the first dose of ipilimumab and rounded down to the nearest integer. Patients' history of systemic cancer therapy was determined by retrospective chart review. A total of 16 patients received systemic cancer treatment, either prior to ipilimumab monotherapy (14/16) or both before and during ipilimumab treatment course (2/16). The symbol (+) indicates treatments that are given concurrently, while symbol (;) indicates treatments administered at different times. The symbol (*) indicates systemic cancer treatment administered both before and during ipilimumab therapy. C-F patients, n=24; PtC patients, n=10.
