# Supplementary material for: Intestinal microbiome analyses identify melanoma patients at risk for checkpoint-blockade-induced colitis
Source: Nat Commun. 2016 Feb 2;7:10391. doi: 10.1038/ncomms10391 (PMC4740747; doi:10.1038/ncomms10391)
Supplement: Supplementary Software — A readme.txt and R code [file ncomms10391-s2.zip › Data/2015-01-23 abundance plots, phylum, samples A_legend, flush-2109.pdf]

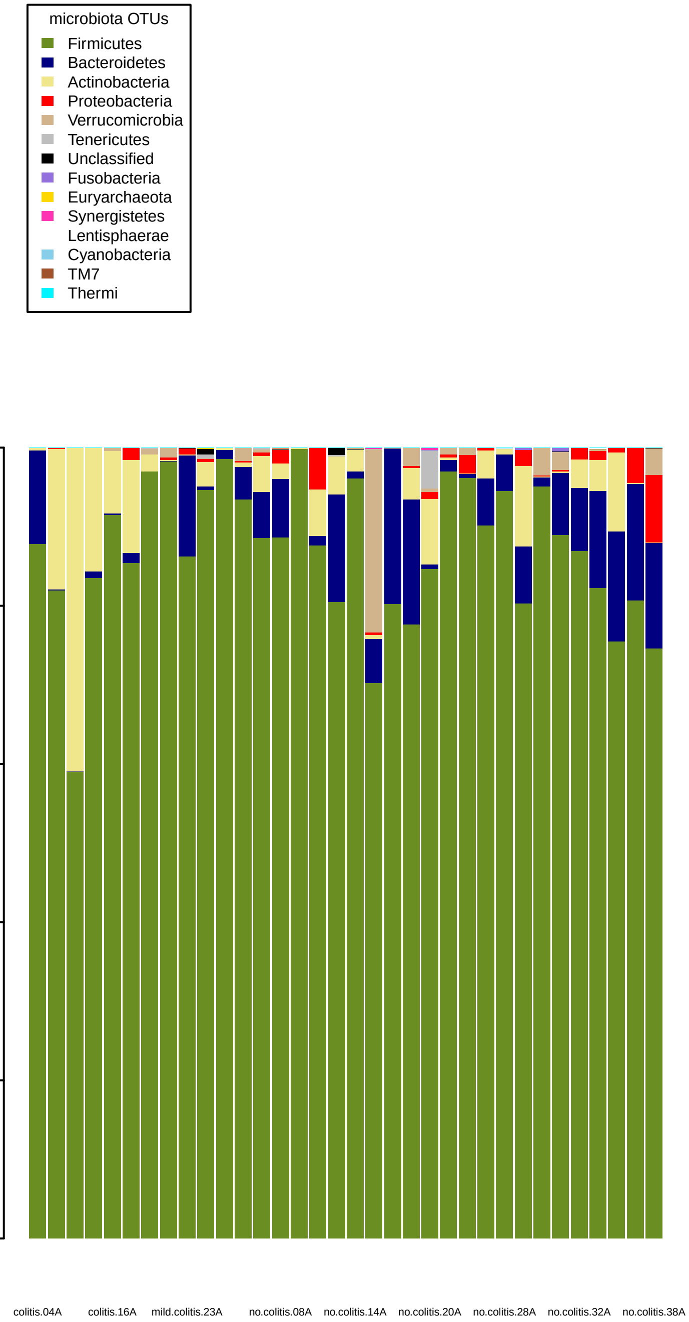
microbiota OTUs
Firmicutes
Bacteroidetes
Actinobacteria
Proteobacteria
Verrucomicrobia
Tenericutes
Unclassified
Fusobacteria
Euryarchaeota
Synergistetes
Lentisphaerae
Cyanobacteria
TM7
Thermi
colitis.04A
colitis.16A
mild.colitis.23A
no.colitis.08A
no.colitis.14A
no.colitis.20A
no.colitis.28A
no.colitis.32A
no.colitis.38A
