# Supplementary material for: Intestinal microbiome analyses identify melanoma patients at risk for checkpoint-blockade-induced colitis
Source: Nat Commun. 2016 Feb 2;7:10391. doi: 10.1038/ncomms10391 (PMC4740747; doi:10.1038/ncomms10391)
Supplement: Supplementary Software — A readme.txt and R code [file ncomms10391-s2.zip › Data/pasted-image-4278.pdf]

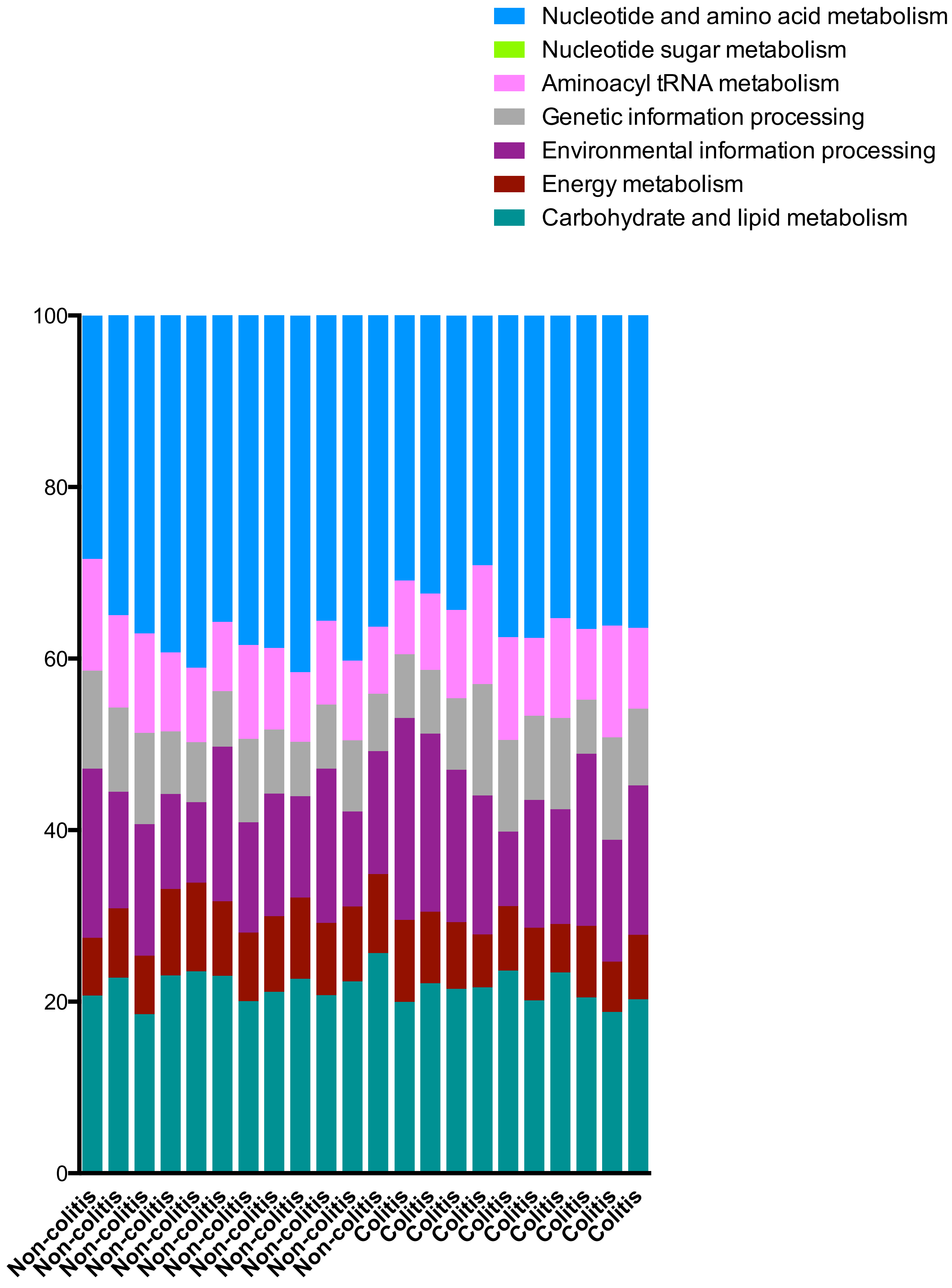
Nucleotide and amino acid metabolism
Nucleotide sugar metabolism
Aminoacyl tRNA metabolism
Genetic information processing
Environmental information processing
Energy metabolism
Carbohydrate and lipid metabolism
100
80
60
40
20
0
Non-colitis
Non-colitis
Non-colitis
Non-colitis
Non-colitis
Non-colitis
Non-colitis
Non-colitis
Non-colitis
Non-colitis
Non-colitis
Colitis
Colitis
Colitis
Colitis
Colitis
Colitis
Colitis
Colitis
Colitis
Colitis
Colitis
