# Supplementary material for: Intestinal microbiome analyses identify melanoma patients at risk for checkpoint-blockade-induced colitis
Source: Nat Commun. 2016 Feb 2;7:10391. doi: 10.1038/ncomms10391 (PMC4740747; doi:10.1038/ncomms10391)
Supplement: Supplementary Software — A readme.txt and R code [file ncomms10391-s2.zip › Data/pasted-image-4282.pdf]

- Nucleotide and amino acid metabolism
- Nucleotide sugar metabolism
- Aminoacyl tRNA metabolism
- Genetic information processing
- Environmental information processing
- Energy metabolism
- Carbohydrate and lipid metabolism
