# Supplementary figures and images for: Intestinal microbiome analyses identify melanoma patients at risk for checkpoint-blockade-induced colitis
Source: Nat Commun. 2016 Feb 2;7:10391. doi: 10.1038/ncomms10391 (PMC4740747; doi:10.1038/ncomms10391)

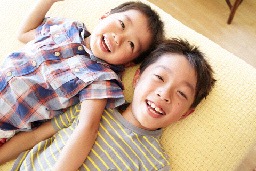

Supplement: Supplementary Software — A readme.txt and R code [file ncomms10391-s2.zip › Data/110809_FamilyChineseOahu_EN_02390_2880x1921-small-10.jpg]

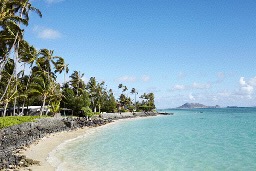

Supplement: Supplementary Software — A readme.txt and R code [file ncomms10391-s2.zip › Data/110809_FamilyChineseOahu_EN_02016_981x654-small-12.jpg]

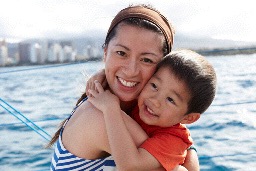

Supplement: Supplementary Software — A readme.txt and R code [file ncomms10391-s2.zip › Data/110809_FamilyChineseOahu_EN_00317_2040x1360-small-14.jpg]

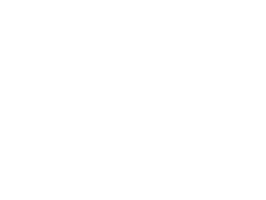

Supplement: Supplementary Software — A readme.txt and R code [file ncomms10391-s2.zip › Data/st0-76.jpg]

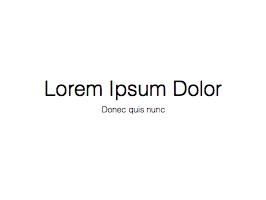

Supplement: Supplementary Software — A readme.txt and R code [file ncomms10391-s2.zip › Data/mt0@2x-77.jpg]

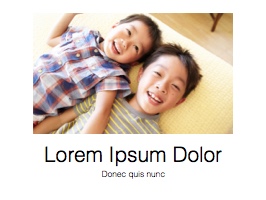

Supplement: Supplementary Software — A readme.txt and R code [file ncomms10391-s2.zip › Data/mt1@2x-78.jpg]

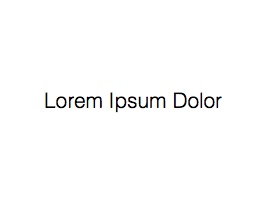

Supplement: Supplementary Software — A readme.txt and R code [file ncomms10391-s2.zip › Data/mt2@2x-79.jpg]

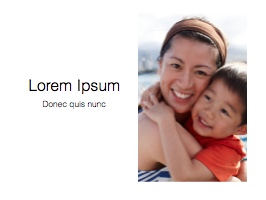

Supplement: Supplementary Software — A readme.txt and R code [file ncomms10391-s2.zip › Data/mt3@2x-80.jpg]

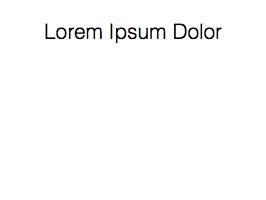

Supplement: Supplementary Software — A readme.txt and R code [file ncomms10391-s2.zip › Data/mt4@2x-81.jpg]

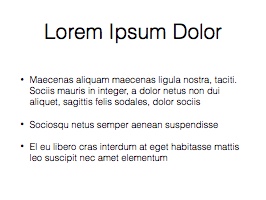

Supplement: Supplementary Software — A readme.txt and R code [file ncomms10391-s2.zip › Data/mt5@2x-82.jpg]

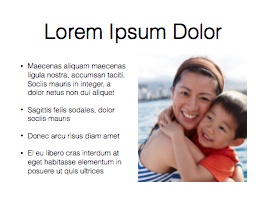

Supplement: Supplementary Software — A readme.txt and R code [file ncomms10391-s2.zip › Data/mt6@2x-83.jpg]

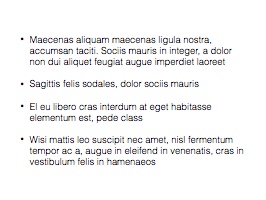

Supplement: Supplementary Software — A readme.txt and R code [file ncomms10391-s2.zip › Data/mt7@2x-84.jpg]

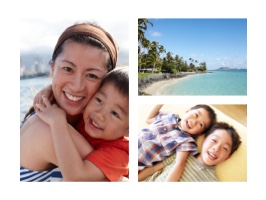

Supplement: Supplementary Software — A readme.txt and R code [file ncomms10391-s2.zip › Data/mt8@2x-85.jpg]

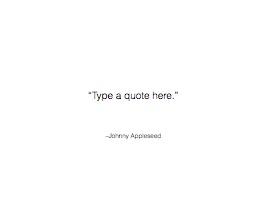

Supplement: Supplementary Software — A readme.txt and R code [file ncomms10391-s2.zip › Data/mt9@2x-86.jpg]

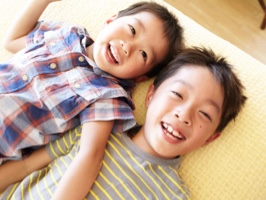

Supplement: Supplementary Software — A readme.txt and R code [file ncomms10391-s2.zip › Data/mt10@2x-87.jpg]

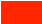

Colitis

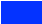

Non-colitis

Supplement: Supplementary Software — A readme.txt and R code [file ncomms10391-s2.zip › Data/pasted-image-708.pdf]

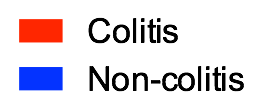

Supplement: Supplementary Software — A readme.txt and R code [file ncomms10391-s2.zip › Data/pasted-image-small-710.png]

Colitis  
Non-colitis

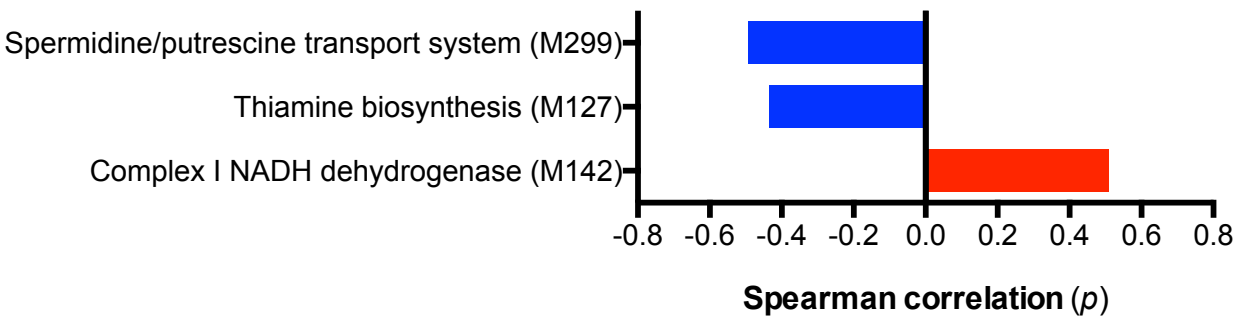

Supplement: Supplementary Software — A readme.txt and R code [file ncomms10391-s2.zip › Data/pasted-image-2058.pdf]

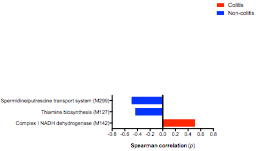

Supplement: Supplementary Software — A readme.txt and R code [file ncomms10391-s2.zip › Data/pasted-image-small-2059.png]

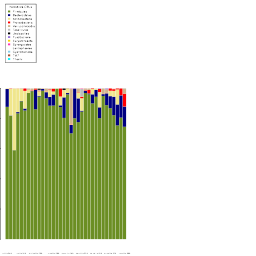

Supplement: Supplementary Software — A readme.txt and R code [file ncomms10391-s2.zip › Data/2015-01-23 abundance plots, phylum, samples A_legend, flush-small-2110.png]

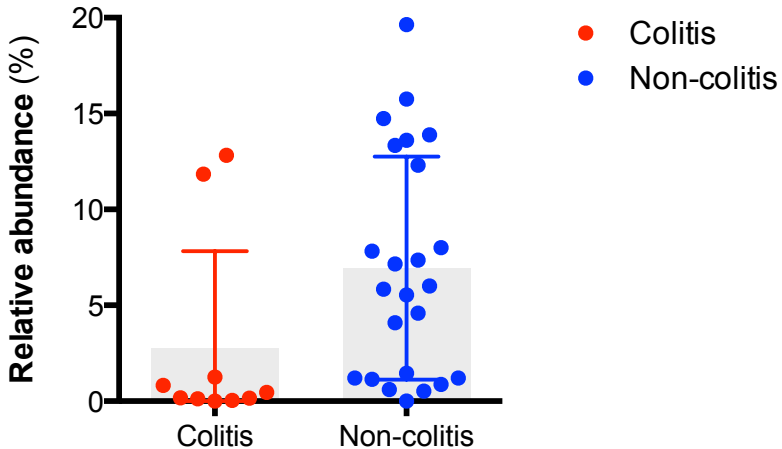

Supplement: Supplementary Software — A readme.txt and R code [file ncomms10391-s2.zip › Data/pasted-image-3363.pdf]

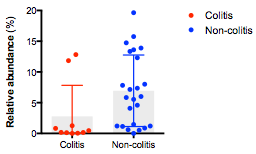

Supplement: Supplementary Software — A readme.txt and R code [file ncomms10391-s2.zip › Data/pasted-image-small-3366.png]

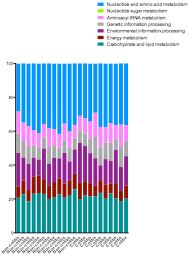

Supplement: Supplementary Software — A readme.txt and R code [file ncomms10391-s2.zip › Data/pasted-image-small-4279.png]

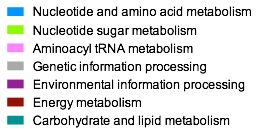

Supplement: Supplementary Software — A readme.txt and R code [file ncomms10391-s2.zip › Data/pasted-image-small-4283.png]

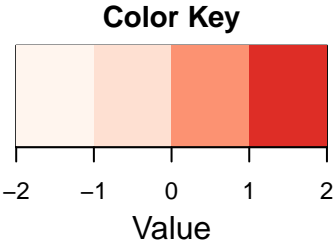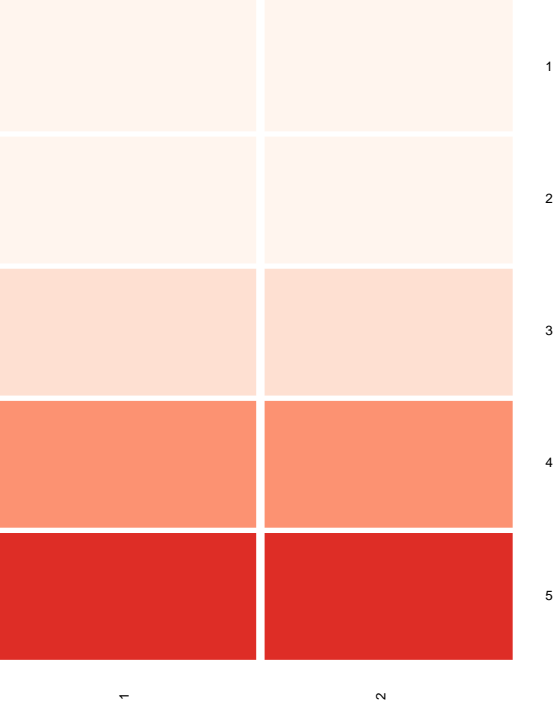

Supplement: Supplementary Software — A readme.txt and R code [file ncomms10391-s2.zip › Data/mapdata key-4418.pdf]

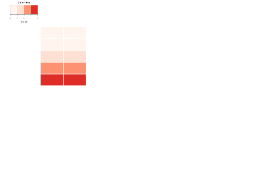

Supplement: Supplementary Software — A readme.txt and R code [file ncomms10391-s2.zip › Data/mapdata key-small-4419.png]

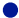

Sample collected

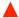

Colitis onset

Supplement: Supplementary Software — A readme.txt and R code [file ncomms10391-s2.zip › Data/pasted-image-4641.pdf]

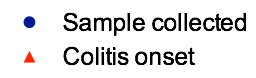

Supplement: Supplementary Software — A readme.txt and R code [file ncomms10391-s2.zip › Data/pasted-image-small-4642.png]

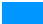

Shared

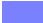

Non-colitis only

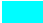

Colitis only

Supplement: Supplementary Software — A readme.txt and R code [file ncomms10391-s2.zip › Data/pasted-image-5662.pdf]

**Non-colits**

Shared  
Remaining

**Colitis**

0 20 40 60 80 100

**Relative abundance (%)**

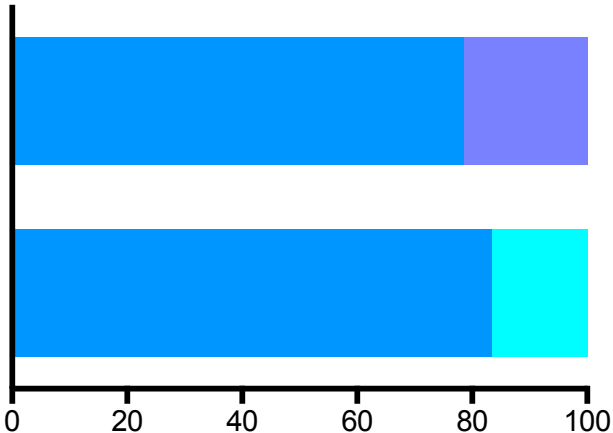

Supplement: Supplementary Software — A readme.txt and R code [file ncomms10391-s2.zip › Data/pasted-image-5663.pdf]

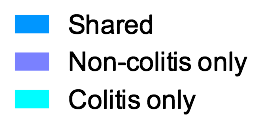

Supplement: Supplementary Software — A readme.txt and R code [file ncomms10391-s2.zip › Data/pasted-image-small-5667.png]

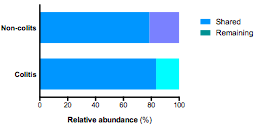

Supplement: Supplementary Software — A readme.txt and R code [file ncomms10391-s2.zip › Data/pasted-image-small-5668.png]

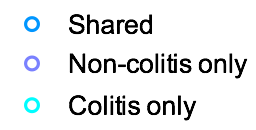

Supplement: Supplementary Software — A readme.txt and R code [file ncomms10391-s2.zip › Data/pasted-image-small-5669.png]

colitis &amp; non-colitis samples: OTU-based heatmap

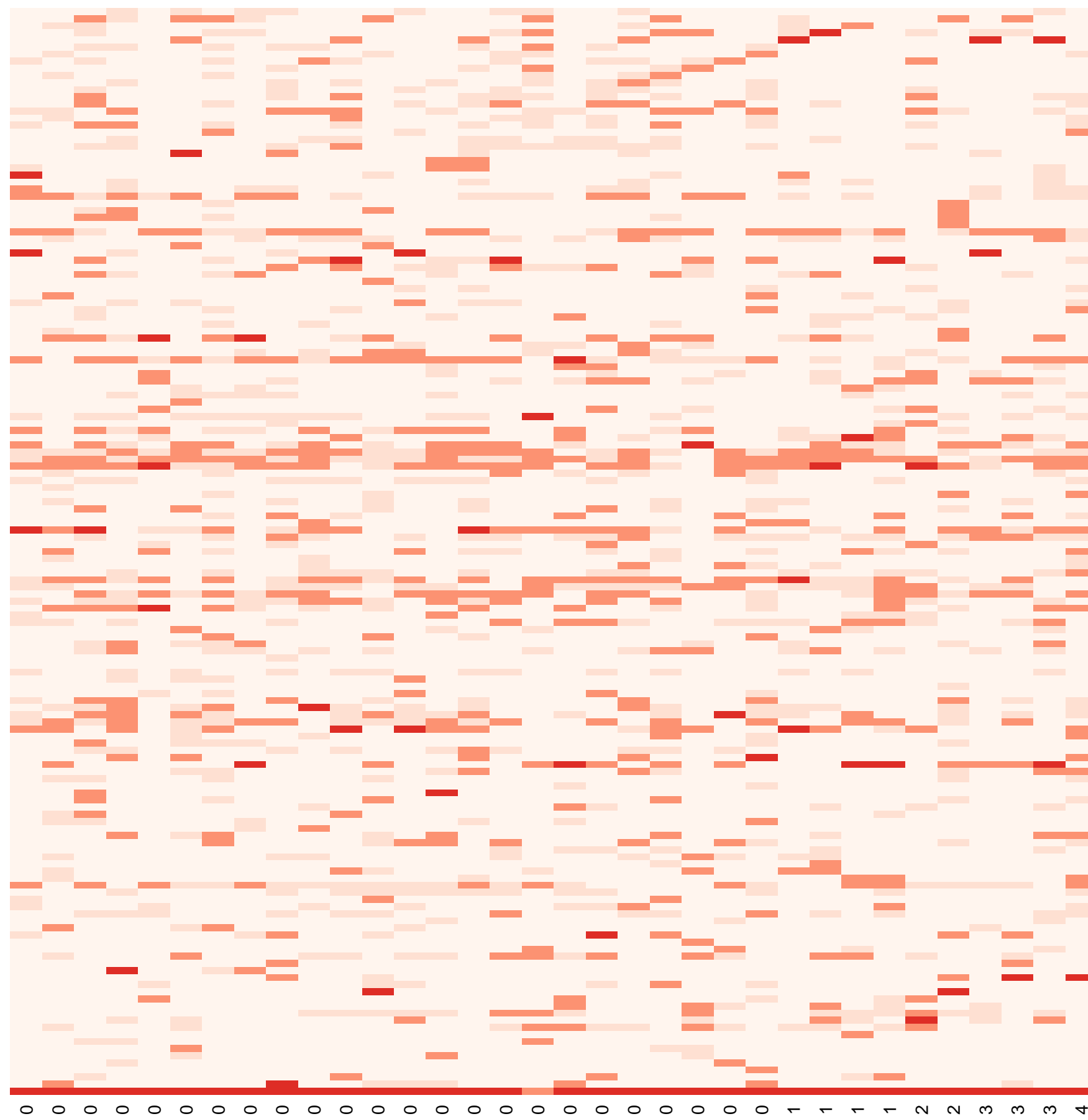[illegible]

Supplement: Supplementary Software — A readme.txt and R code [file ncomms10391-s2.zip › Data/Ipi heatmap, log, colored background, 2.22.15, seashell-5860.pdf]

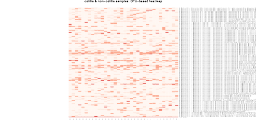

Supplement: Supplementary Software — A readme.txt and R code [file ncomms10391-s2.zip › Data/Ipi heatmap, log, colored background, 2.22-5861.png]

# Clinical metadata

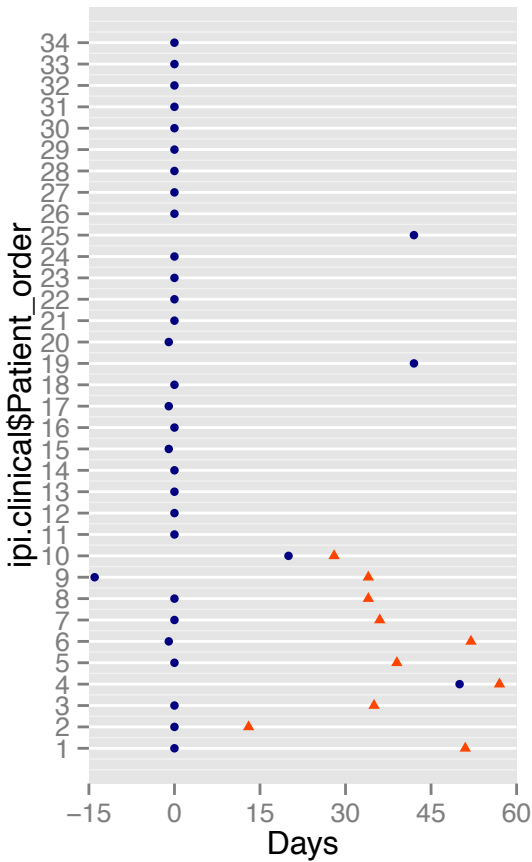

Supplement: Supplementary Software — A readme.txt and R code [file ncomms10391-s2.zip › Data/Ipi timeline, 3x5, 3.10.15-6111.pdf]

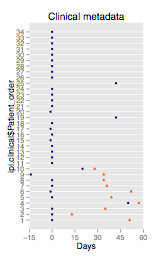

Supplement: Supplementary Software — A readme.txt and R code [file ncomms10391-s2.zip › Data/Ipi timeline, 3x5, 3.10-6112.png]

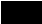

Sensitivity

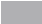

Specificity

Supplement: Supplementary Software — A readme.txt and R code [file ncomms10391-s2.zip › Data/pasted-image-7934.pdf]

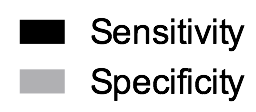

Supplement: Supplementary Software — A readme.txt and R code [file ncomms10391-s2.zip › Data/pasted-image-small-7935.png]

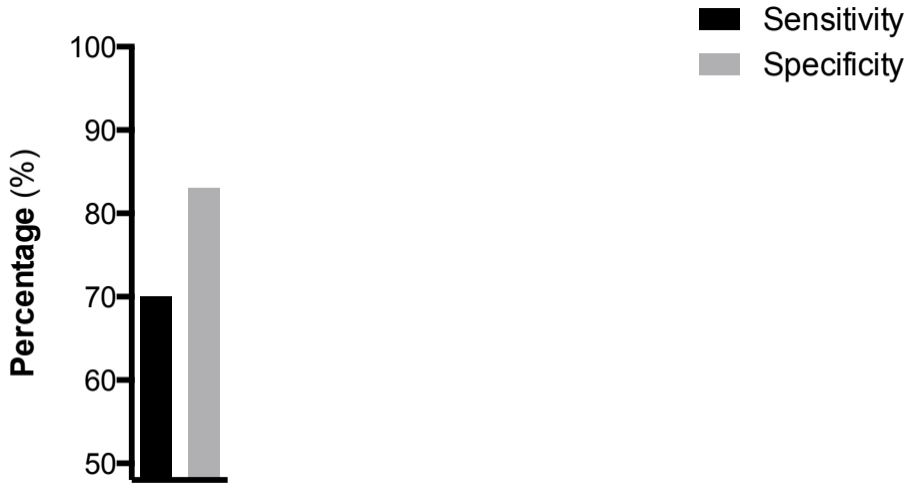

Supplement: Supplementary Software — A readme.txt and R code [file ncomms10391-s2.zip › Data/pasted-image-8392.pdf]

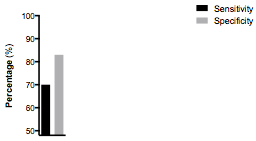

Supplement: Supplementary Software — A readme.txt and R code [file ncomms10391-s2.zip › Data/pasted-image-small-8393.png]

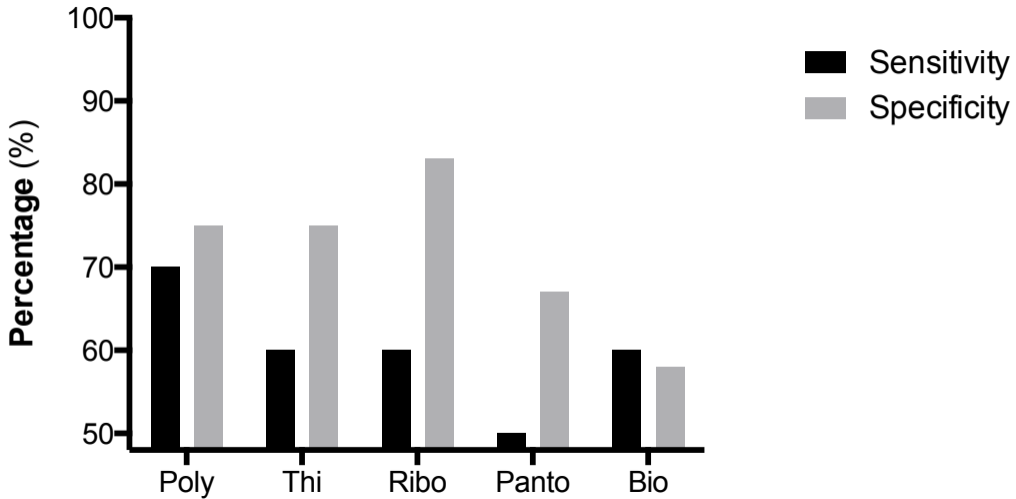

Supplement: Supplementary Software — A readme.txt and R code [file ncomms10391-s2.zip › Data/pasted-image-10710.pdf]

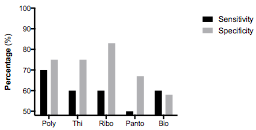

Supplement: Supplementary Software — A readme.txt and R code [file ncomms10391-s2.zip › Data/pasted-image-small-10711.png]

**Number of patients**

**Colitis**

**Non-colitis**

15

10

5

0

**Colitis node**

**No colitis node**

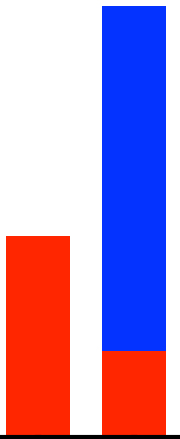

Supplement: Supplementary Software — A readme.txt and R code [file ncomms10391-s2.zip › Data/pasted-image-11820.pdf]

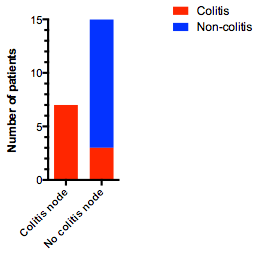

Supplement: Supplementary Software — A readme.txt and R code [file ncomms10391-s2.zip › Data/pasted-image-small-11821.png]

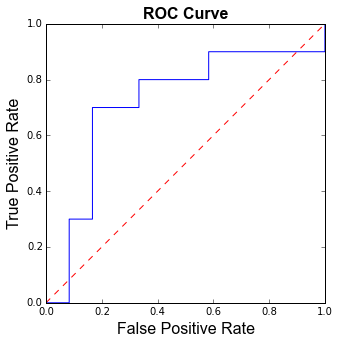

Supplement: Supplementary Software — A readme.txt and R code [file ncomms10391-s2.zip › Data/ROC curve 4 modules, 10.8.15, square-13086.png]

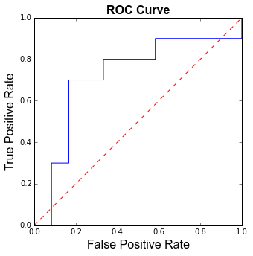

Supplement: Supplementary Software — A readme.txt and R code [file ncomms10391-s2.zip › Data/ROC curve 4 modules, 10.8-13087.png]

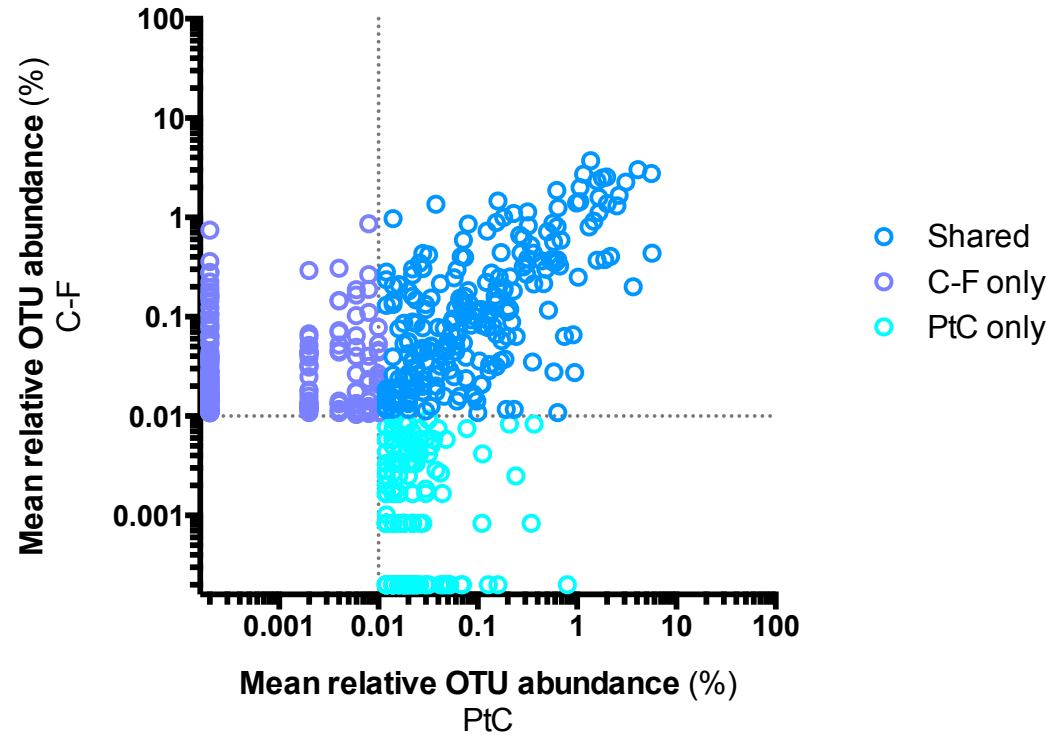

Supplement: Supplementary Software — A readme.txt and R code [file ncomms10391-s2.zip › Data/pasted-image-14284.pdf]

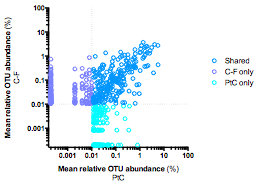

Supplement: Supplementary Software — A readme.txt and R code [file ncomms10391-s2.zip › Data/pasted-image-small-14285.png]

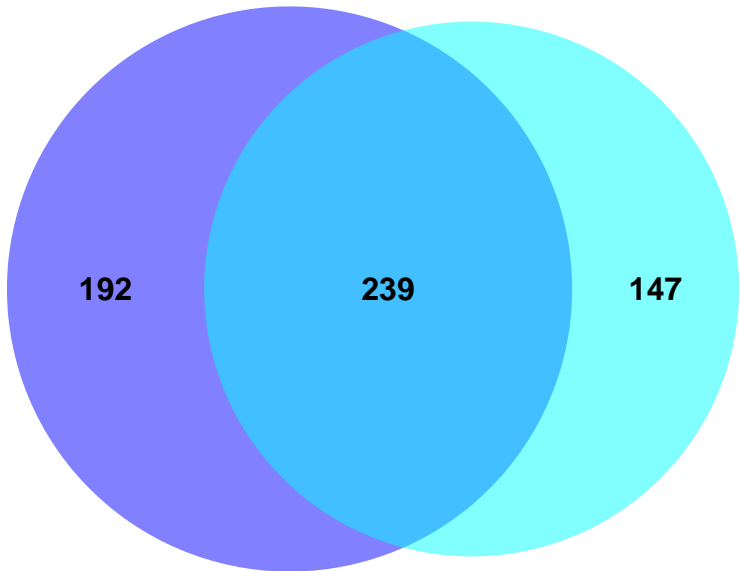

Supplement: Supplementary Software — A readme.txt and R code [file ncomms10391-s2.zip › Data/venn-14327.pdf]

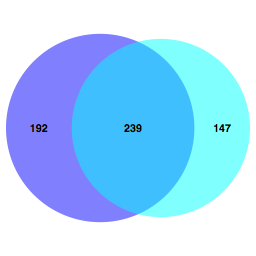

Supplement: Supplementary Software — A readme.txt and R code [file ncomms10391-s2.zip › Data/venn-small-14328.png]

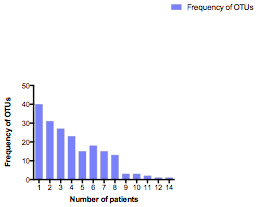

Supplement: Supplementary Software — A readme.txt and R code [file ncomms10391-s2.zip › Data/pasted-image-small-14373.png]

Frequency of OTUs

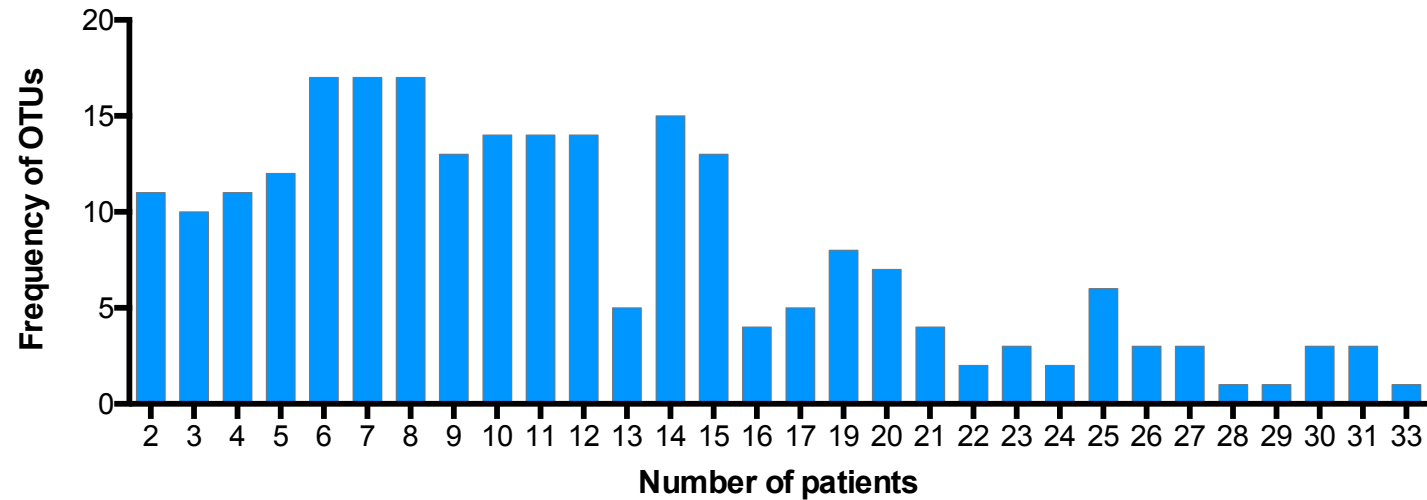

Supplement: Supplementary Software — A readme.txt and R code [file ncomms10391-s2.zip › Data/pasted-image-14408.pdf]

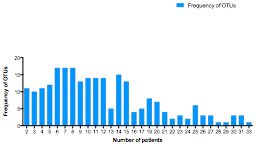

Supplement: Supplementary Software — A readme.txt and R code [file ncomms10391-s2.zip › Data/pasted-image-small-14409.png]

Frequency of OTUs

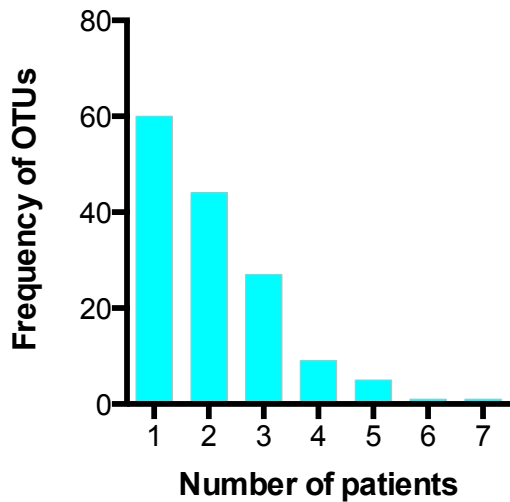

Supplement: Supplementary Software — A readme.txt and R code [file ncomms10391-s2.zip › Data/pasted-image-14412.pdf]

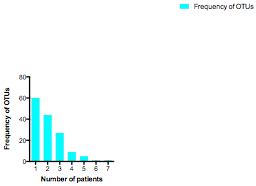

Supplement: Supplementary Software — A readme.txt and R code [file ncomms10391-s2.zip › Data/pasted-image-small-14413.png]

Frequency of OTUs

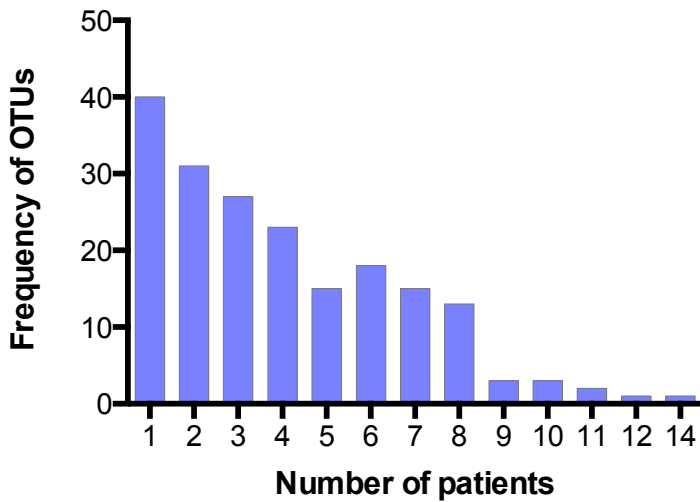

Supplement: Supplementary Software — A readme.txt and R code [file ncomms10391-s2.zip › Data/pasted-image-14416.pdf]

■ C-F

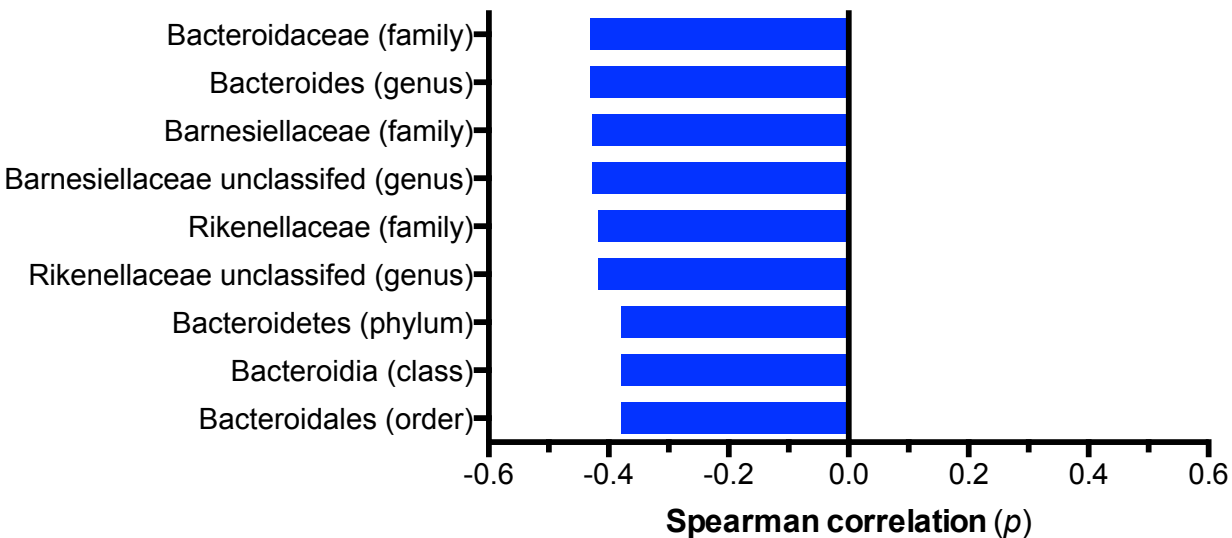

Supplement: Supplementary Software — A readme.txt and R code [file ncomms10391-s2.zip › Data/pasted-image-14499.pdf]

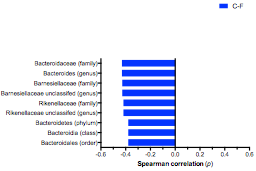

Supplement: Supplementary Software — A readme.txt and R code [file ncomms10391-s2.zip › Data/pasted-image-small-14500.png]

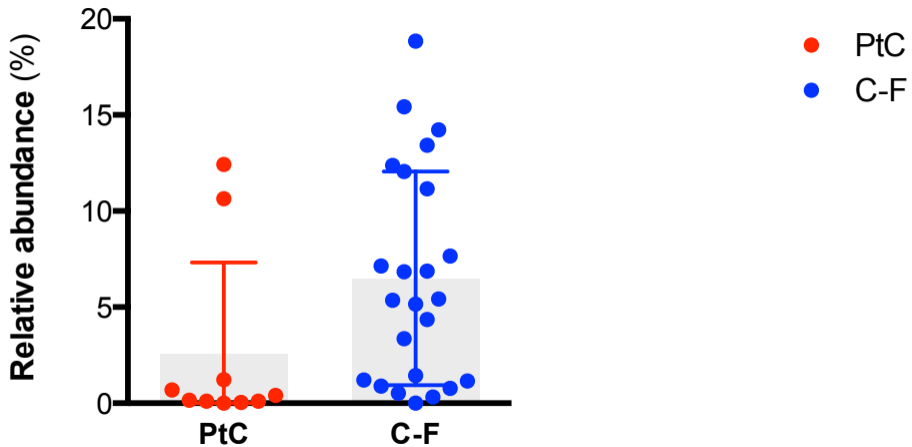

Supplement: Supplementary Software — A readme.txt and R code [file ncomms10391-s2.zip › Data/pasted-image-14541.pdf]

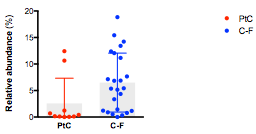

Supplement: Supplementary Software — A readme.txt and R code [file ncomms10391-s2.zip › Data/pasted-image-small-14542.png]

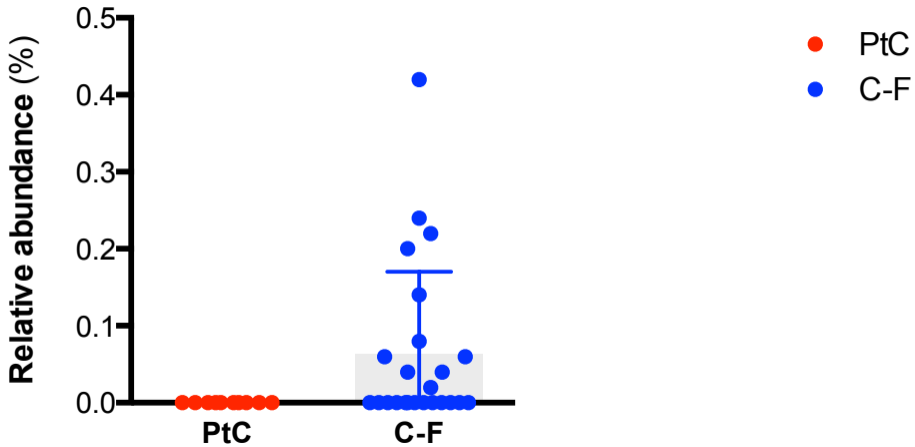

Supplement: Supplementary Software — A readme.txt and R code [file ncomms10391-s2.zip › Data/pasted-image-14549.pdf]

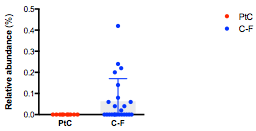

Supplement: Supplementary Software — A readme.txt and R code [file ncomms10391-s2.zip › Data/pasted-image-small-14550.png]

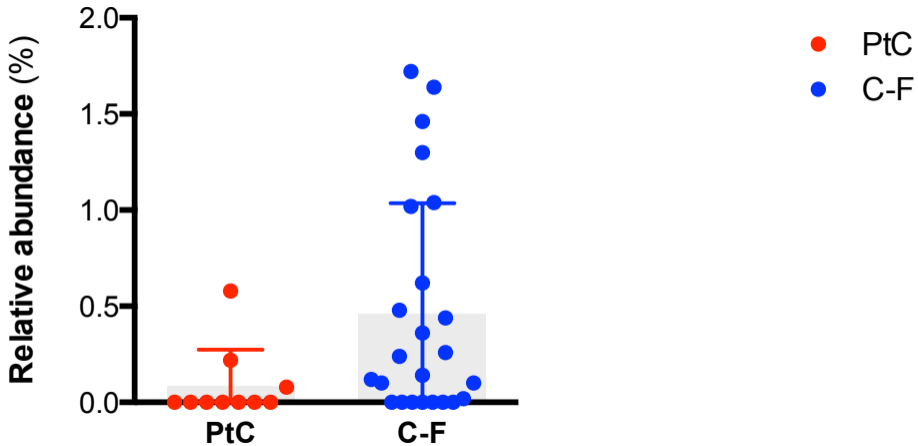

Supplement: Supplementary Software — A readme.txt and R code [file ncomms10391-s2.zip › Data/pasted-image-14557.pdf]

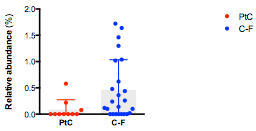

Supplement: Supplementary Software — A readme.txt and R code [file ncomms10391-s2.zip › Data/pasted-image-small-14558.png]

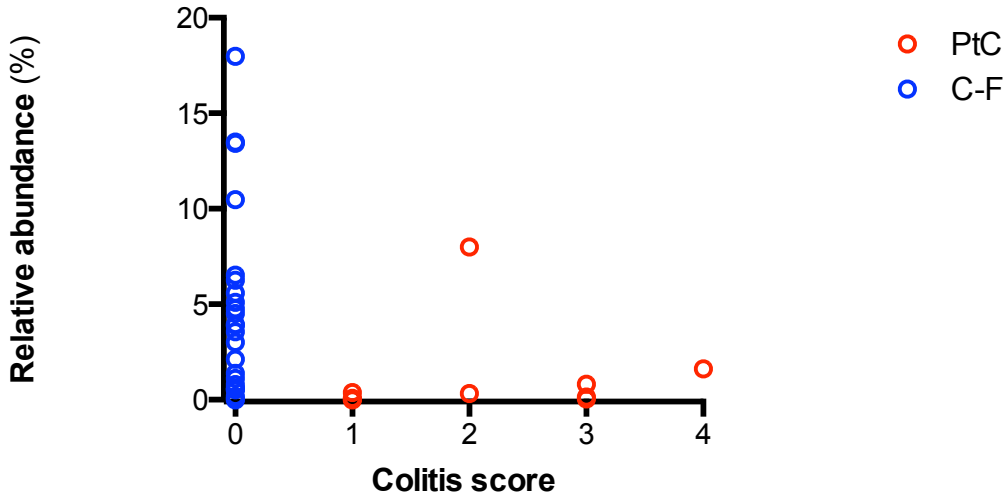

Supplement: Supplementary Software — A readme.txt and R code [file ncomms10391-s2.zip › Data/pasted-image-14588.pdf]

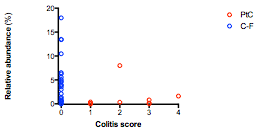

Supplement: Supplementary Software — A readme.txt and R code [file ncomms10391-s2.zip › Data/pasted-image-small-14589.png]

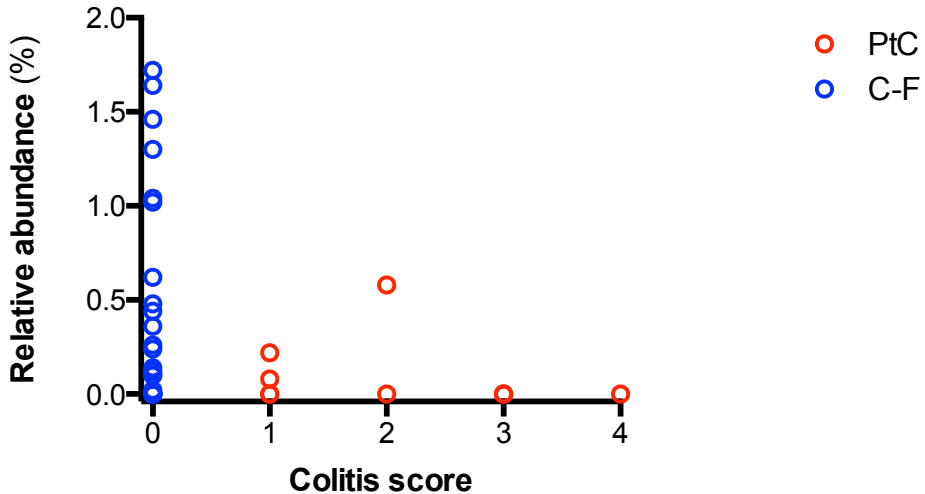

Supplement: Supplementary Software — A readme.txt and R code [file ncomms10391-s2.zip › Data/pasted-image-14595.pdf]

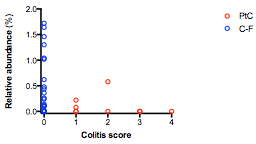

Supplement: Supplementary Software — A readme.txt and R code [file ncomms10391-s2.zip › Data/pasted-image-small-14596.png]

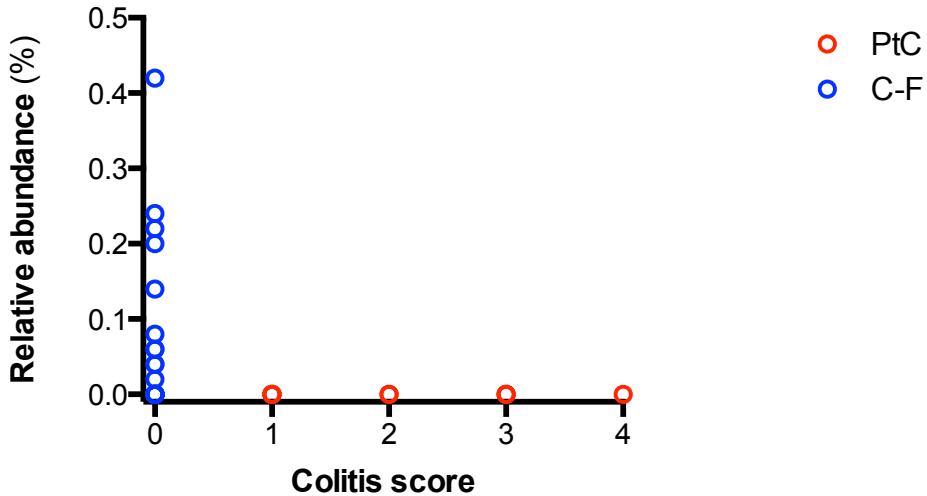

Supplement: Supplementary Software — A readme.txt and R code [file ncomms10391-s2.zip › Data/pasted-image-14601.pdf]

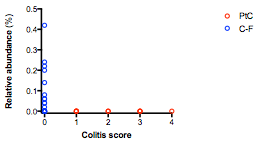

Supplement: Supplementary Software — A readme.txt and R code [file ncomms10391-s2.zip › Data/pasted-image-small-14602.png]

No. of OTUs  
Bacteroidetes phylum

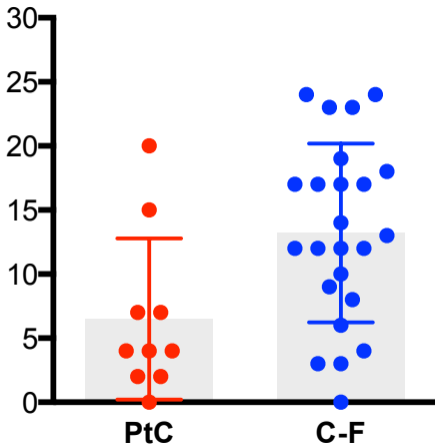

● PtC  
● C-F

Supplement: Supplementary Software — A readme.txt and R code [file ncomms10391-s2.zip › Data/pasted-image-14675.pdf]

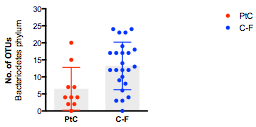

Supplement: Supplementary Software — A readme.txt and R code [file ncomms10391-s2.zip › Data/pasted-image-small-14676.png]

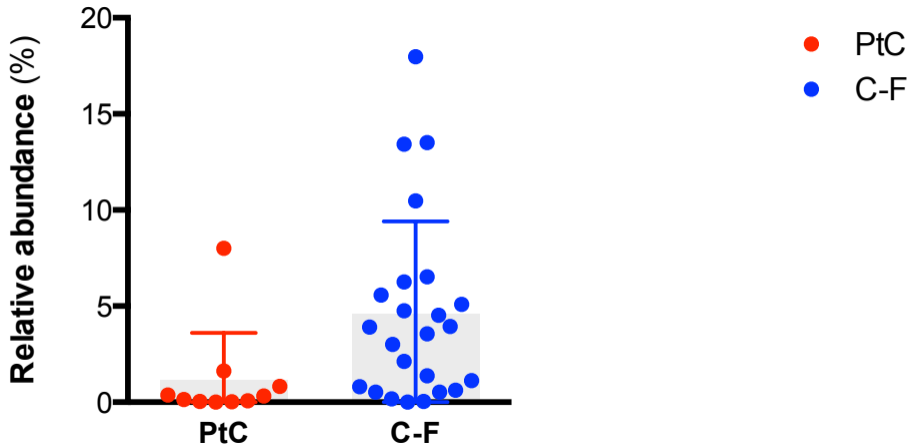

Supplement: Supplementary Software — A readme.txt and R code [file ncomms10391-s2.zip › Data/pasted-image-14702.pdf]

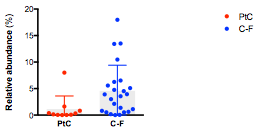

Supplement: Supplementary Software — A readme.txt and R code [file ncomms10391-s2.zip › Data/pasted-image-small-14703.png]

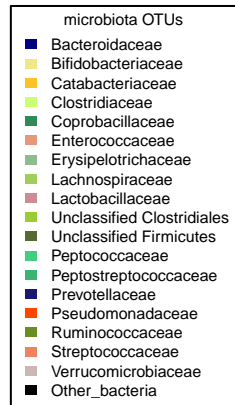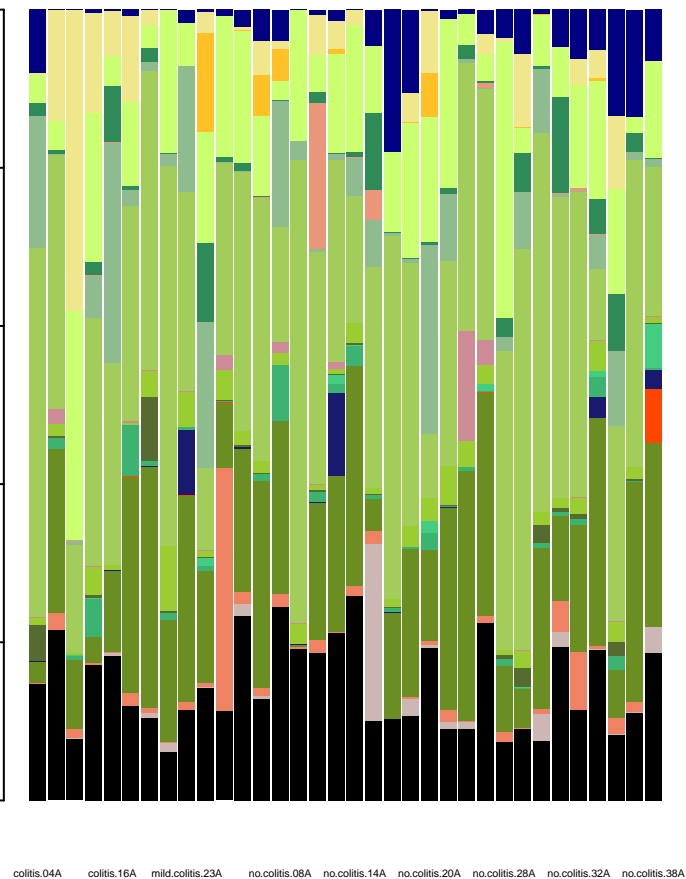

Supplement: Supplementary Software — A readme.txt and R code [file ncomms10391-s2.zip › Data/2015-10-14 abundance plots, family, trimmed, samples A, updated color palette final_legend, flush, better orientation-14957.pdf]

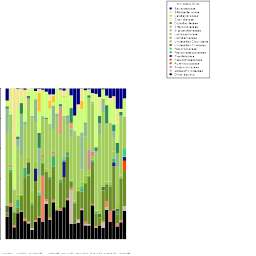

Supplement: Supplementary Software — A readme.txt and R code [file ncomms10391-s2.zip › Data/2015-10-14 abundance plots, family, trimmed, samples A, updated color palette final_legend, flush, better orientation-small-14958.png]

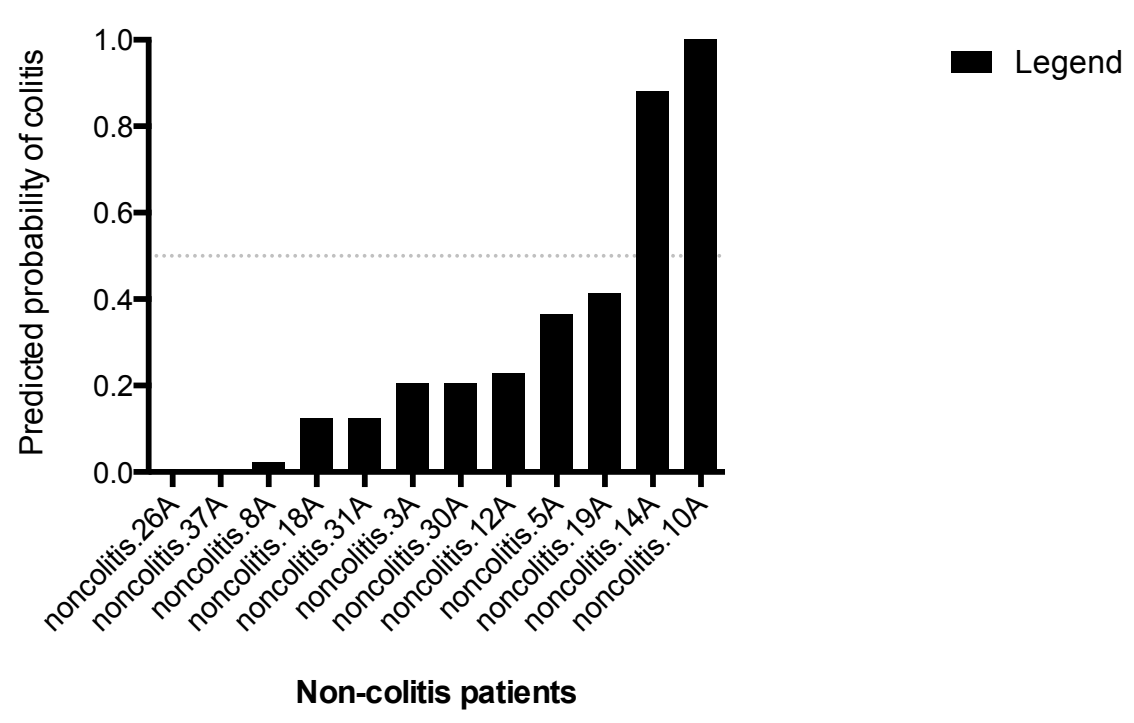

Supplement: Supplementary Software — A readme.txt and R code [file ncomms10391-s2.zip › Data/pasted-image-16001.pdf]

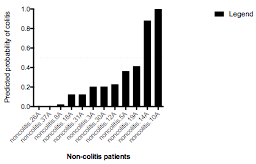

Supplement: Supplementary Software — A readme.txt and R code [file ncomms10391-s2.zip › Data/pasted-image-small-16002.png]

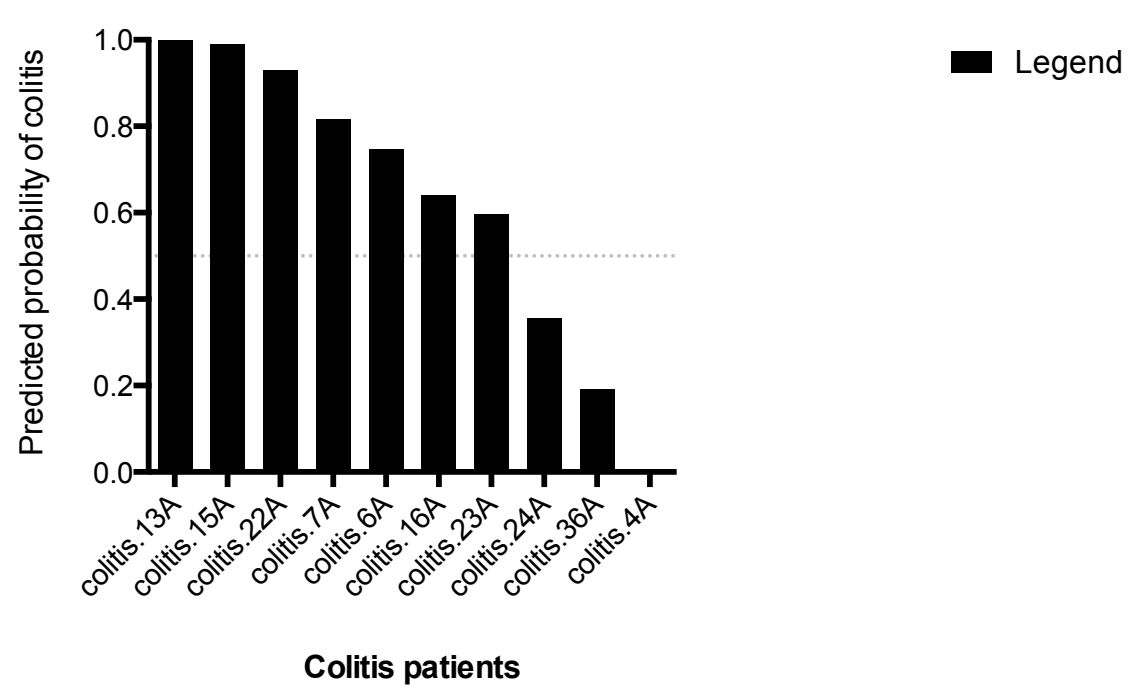

Supplement: Supplementary Software — A readme.txt and R code [file ncomms10391-s2.zip › Data/pasted-image-16005.pdf]

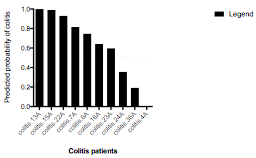

Supplement: Supplementary Software — A readme.txt and R code [file ncomms10391-s2.zip › Data/pasted-image-small-16006.png]

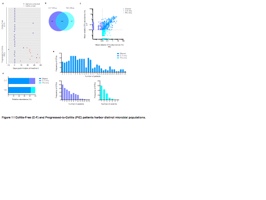

Supplement: Supplementary Software — A readme.txt and R code [file ncomms10391-s2.zip › Data/st0-17478.jpg]

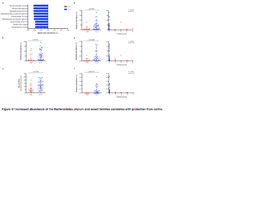

Supplement: Supplementary Software — A readme.txt and R code [file ncomms10391-s2.zip › Data/st2-17480.jpg]

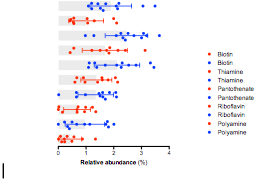

Supplement: Supplementary Software — A readme.txt and R code [file ncomms10391-s2.zip › Data/pasted-image-small-17490.png]

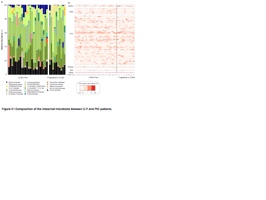

Supplement: Supplementary Software — A readme.txt and R code [file ncomms10391-s2.zip › Data/st1-17566.jpg]

Non-colitis  
Colitis

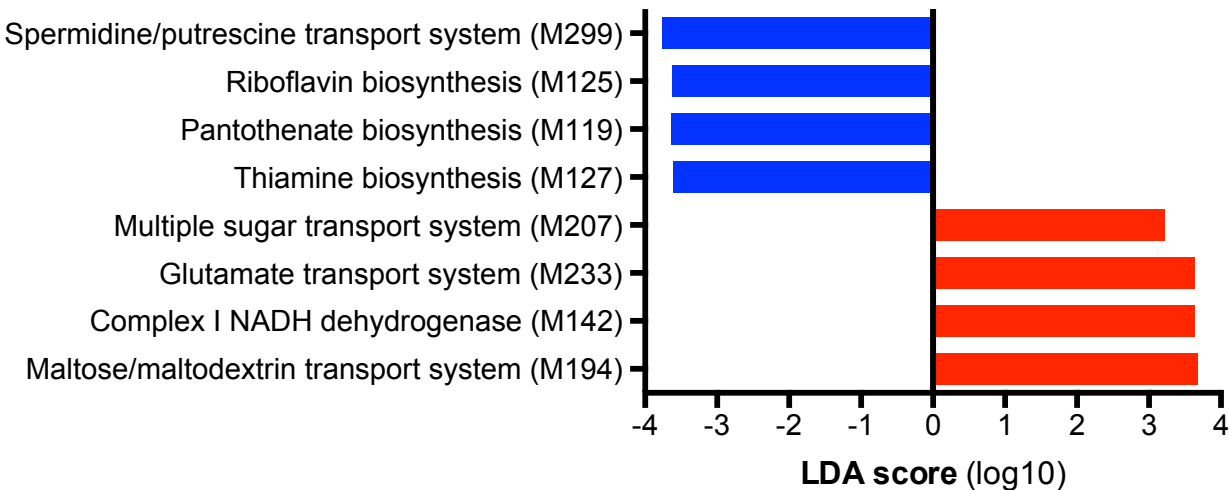

Supplement: Supplementary Software — A readme.txt and R code [file ncomms10391-s2.zip › Data/pasted-image-17567.pdf]

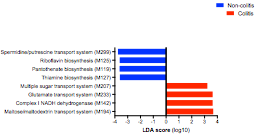

Supplement: Supplementary Software — A readme.txt and R code [file ncomms10391-s2.zip › Data/pasted-image-small-17568.png]

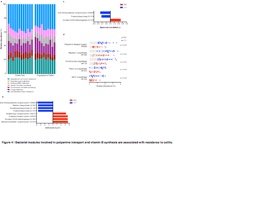

Supplement: Supplementary Software — A readme.txt and R code [file ncomms10391-s2.zip › Data/st-CE2709AF-DE2D-48A2-90C4-E005890A285B-17846.jpg]

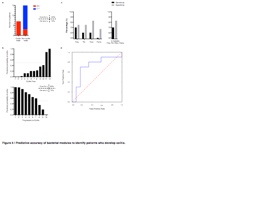

Supplement: Supplementary Software — A readme.txt and R code [file ncomms10391-s2.zip › Data/st-C1EA9A28-46C5-420D-B87C-66677DE575D2-18465.jpg]

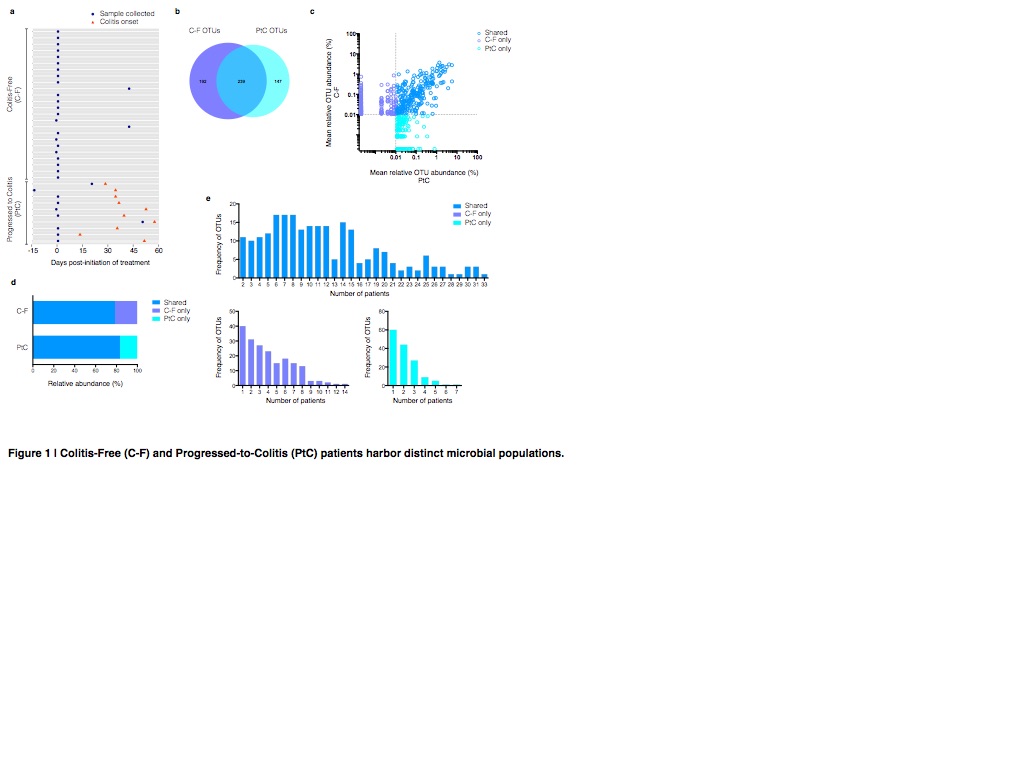

Supplement: Supplementary Software — A readme.txt and R code [file ncomms10391-s2.zip › preview.jpg]

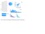

Supplement: Supplementary Software — A readme.txt and R code [file ncomms10391-s2.zip › preview-micro.jpg]

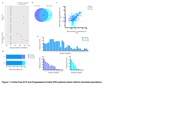

Supplement: Supplementary Software — A readme.txt and R code [file ncomms10391-s2.zip › preview-web.jpg]
